# Functionalized Carbon Nanotubes for Delivery of Ferulic Acid and Diosgenin Anticancer Natural Agents

**DOI:** 10.1021/acsabm.3c00700

**Published:** 2024-01-22

**Authors:** Khaled AbouAitah, Ahmed M. Abdelaziz, Imane M. Higazy, Anna Swiderska-Sroda, Abeer M. E. Hassan, Olfat G. Shaker, Urszula Szałaj, Leszek Stobinski, Artur Malolepszy, Witold Lojkowski

**Affiliations:** † Medicinal and Aromatic Plants Research Department, Pharmaceutical and Drug Industries Research Institute, National Research Centre (NRC), 33 El-Behouth Street, Dokki, Giza 12622, Egypt; ‡ Supplementary General Sciences, Future University, End of 90th Street, Fifth Settlement, New Cairo 11835, Egypt; § Department of Pharmaceutical Technology, Pharmaceutical and Drug Industries Research Institute, National Research Centre (NRC), 33 El-Behouth Street, Dokki, Giza 12622, Egypt; ∥ Institute of High Pressure Physics, Polish Academy of Sciences, Sokolowska 29/37, 01-142 Warsaw, Poland; ⊥ Analytical Chemistry Department, Faculty of Pharmacy, October 6 University, Giza 12585, Egypt; # Medical Biochemistry and Molecular Biology Department, Faculty of Medicine, Cairo University, Cairo 11511, Egypt; ¶ Faculty of Materials Engineering, Warsaw University of Technology, Wołoska 41, 02-507 Warsaw, Poland; ∇ NANOMATPL Ltd., 14/38 Wyszogrodzka Street, Warsaw 03-337, Poland; ○ Faculty of Chemical and Process Engineering, Warsaw University of Technology, 1 Warynskiego Street, 00-645 Warsaw, Poland

**Keywords:** carbon nanotubes, codelivery system, diosgenin and ferulic acid, kinetic release, cancer cells, long noncoding RNAs/microRNAs/TGF-β and *E*-cadherin protein

## Abstract

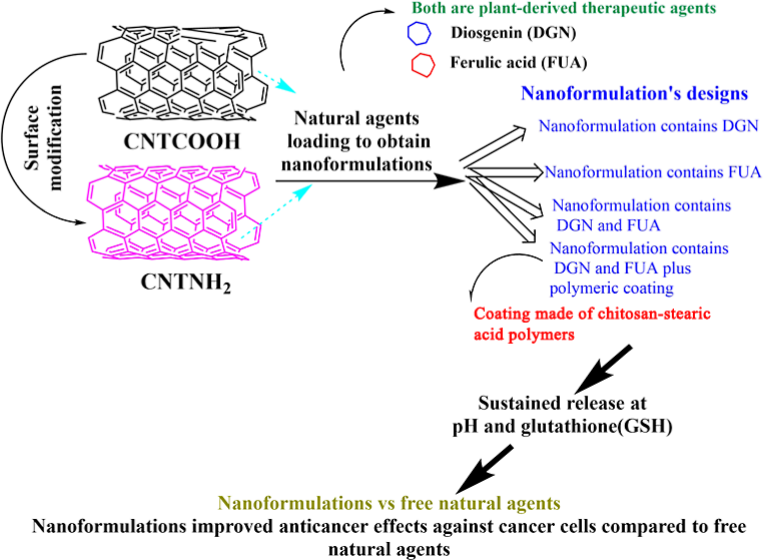

It was investigated whether loading multi-wall carbon nanotubes (CNTs) with two natural anticancer agents: ferulic acid (FUA) and diosgenin (DGN), may enhance the anticancer effect of these drugs. The CNTs were functionalized with carboxylic acid (CNTCOOH) or amine (CNTNH_2_), loaded with the above pro-drugs, as well as both combined and coated with chitosan or chitosan–stearic acid. Following physicochemical characterization, the drug-loading properties and kinetics of the drug’s release were investigated. Their effects on normal human skin fibroblasts and MCF-7 breast carcinoma cells, HepG2 hepatocellular carcinoma cells, and A549 non-small-cell lung cancer cells were evaluated in vitro. Their actions at the molecular level were evaluated by assessing the expression of lncRNAs (HULC, HOTAIR, CCAT-2, H19, and HOTTIP), microRNAs (mir-21, mir-92, mir-145, and mir-181a), and proteins (TGF-β and *E*-cadherin) in HepG2 cells. The release of both pro-drugs depended on the glutathione concentration, coating, and functionalization. Release occurred in two stages: a no-burst/zero-order release followed by a sustained release best fitted to Korsmeyer–Peppas kinetics. The combined nanoformulation cancer inhibition effect on HepG2 cancer cells was more pronounced than for A549 and MCF7 cells. The combined nanoformulations had an additive impact followed by a synergistic effect, with antagonism demonstrated at high concentrations. The nanoformulation coated with chitosan and stearic acid was particularly successful in targeting HepG2 cells and inducing apoptosis. The CNT functionalized with carboxylic acid (CNTCOOH), loaded with both FUA and DGN, and coated with chitosan–stearic acid inhibited the expression of lncRNAs and modulated both microRNAs and proteins. Thus, nanoformulations composed of functionalized CNTs dual-loaded with FUA and DGN and coated with chitosan–stearic acid are a promising drug delivery system that enhances the activity of natural pro-drugs.

## 1 Introduction

A major challenge in treating cancer is precisely and specifically targeting the site where the drug or therapeutic agent is required^1^ to increase treatment efficiency and reduce negative side effects. Therefore, efficient targeted drug delivery systems (DDSs) to deliver the drug to the cancer site and allow controlled release have been extensively researched. In recent years, DDSs have emerged as effective strategies in cancer treatment.

DDS based on nanoparticles is an important branch of research within the general field of nanomedicine—i.e., the application of nanotechnology in medicine.^2^ The underlying idea is that nanoparticles may have a high drug-loading capacity and may penetrate cells. Many nanomaterials have been utilized as vehicles for DDSs. Among these nanomaterials, carbon nanotubes (CNTs) have garnered much attention as a favorable nanocarrier due to their favorable physicochemical properties: shape, high surface area, chemical stability, possibility of multiple surface modifications, high drug-loading capacity, and other properties.^3−6^ CNTs may penetrate cell membranes and be internalized into cells.^7,8^ The flexibility, length, and diameter of CNTs greatly differ depending on how they are manufactured and applied.^9^ These properties are of great importance in biomedical applications as they determine cytotoxicity and biosafety^9,10^ and can affect cellular uptake processes.^11^

The CNTs have a great potential compared to other drug carriers, like liposomes and polymeric ones, as functionalization of their surfaces (due to their hexagonal close-packed cylindrical structure)^12^ and attachment of active molecules is relatively easy. Further, their specific surface area (SSA) is remarkably high, so a high loading capacity is expected. The possibility of controlling their length offers a new dimension in their structuring. Also, drug delivery with liposomes is limited due to the inherent problems: low encapsulation efficiency, low solubility, rapid leakage for water-soluble therapeutics, and storage problems.^13^ Also, the multi-walled CNTs (MWCNTs) may overcome the limitations of polymeric NPs, such as premature drug release and uncontrollable nanoformulation’s preparation.

There are three kinds of CNTs: single-walled CNTs, double-walled CNTs, and MWCNTs. We will focus on MWCNTs as they are widely explored in DDSs to control and deliver various anticancer chemotherapeutic drugs and are most facile to produce. For example, Leyva-González et al.^14^ loaded hyaluronic acid-functionalized MWCNTs with carboplatin to enhance the anticancer activity compared to a free anticancer drug. They tested them in vitro in HeLa and MDA-MB-231 cells (cervical and mammary adenocarcinomas, respectively). Yoong et al.^15^ showed in several cancer cell lines that MWCNTs functionalized to target mitochondria and further encapsulated with cisplatin platinum(IV) exhibit superior efficacy over free cisplatin.

In the present study, we focus on the DDS application for natural pro-drug delivery. These natural anticancer agents have been extensively investigated due to their potential safety, cost-effectiveness, and variety of pharmacological activities. Despite these possible advantages, there are barriers for their clinical use due to their poor bioavailability, poor water solubility, inability to target a specific site, and uncontrolled release kinetics.

Phyto steroidal saponin diosgenin (DGN) is a major bioactive compound found in *Trigonella foenum-graecum* (fenugreek) seeds and *Dioscorea villosa* (wild yam) roots. DGN inhibits tumor cell proliferation and induces apoptosis in the colon, prostate, liver, breast, osteosarcoma, and leukemia.^16,17^ The primary anticancer mechanism of DGN is the modulation of interconnected cell signaling pathways, permitting cell cycle regulation, differentiation, and apoptosis.^18^ DGN is extensively used to manufacture multiple synthetic steroidal drugs for different purposes.^19^ Derivatives of DGN are promising new antitumor agents.^20^

Attempts have recently been made to further improve the anticancer effects of DGN by using niosomes,^21^ eight-arm-polyethylene glycol–DGN conjugate,^22^ and polymeric nanoparticles^23^ as DDS.

Ferulic acid (FUA), a derivative of 4-hydroxycinnamic acid, is a naturally occurring compound found in rice, wheat, oats, grains, fruits, beverages, and medicinal plants. FUA has antioxidant, anti-inflammatory, and antibacterial properties, making it important in the treatment of neurodegenerative diseases, diabetes, cardiovascular disease, inflammation, bacterial infections, and viral infections.^24^ FUA has demonstrated a significant impact against cervical carcinoma, colon, prostate, liver, breast, and pancreatic cancer.^25−27^ Recent studies have demonstrated nanobased DDS for FUA for cancer therapy with chitosan-coated folic acid–solid lipid NPs, PLGA NPs, and nanostructured lipid carriers.^28−30^

In the present paper, we explore whether MWCNTs can be used as a nano-DDS for DGN or FUA.

## 2 Materials and Methods

All chemicals and materials were used in this study without further purification.

### 2.1 Surface Modification of CNTs

Surface modification of CNTs was performed to create functional groups to enhance pro-drug attachment during the loading process. Carboxylic acid functionalization (COOH) and amino groups (–NH_2_) were well-known as surface modifications for CNT.^31,32^ Carboxylic acid-functionalized MWCNTs were synthesized, as described in a previous paper.^33^ They were designated as CNTCOOH. To obtain CNTs functionalized with amino groups (–NH_2_), 3-aminopropyltriethoxysilane (APTES; Sigma-Aldrich, St. Louis, MO, USA) was used. We suspended 1 g of CNTCOOH in 50 mL of anhydrous toluene (POCH, Poland) with sonication (Elma GmbH, Singen, Germany) at room temperature (RT) for 5 min. Subsequently, 2 mL of APTES was slowly dropped into this solution within 5 min and left to stir at 450 rpm for 24 h at RT. We collected the resulting material by centrifugation at 10,000 rpm for 10 min (Cooling Sigma 16K, Laborzentrifugen GmbH, Osterode am Harz, Germany). The collected material was washed three times with methanol, oven-dried at 50 °C for 6 h, and labeled as CNTNH_2_.

### 2.2 DGN and FUA Loading

We applied a drug/nanocarrier ratio of 1/3 by weight. We loaded the DGN and/or FUA via the following steps. 100 mg of DGN or 100 mg of FUA was dissolved in 10 mL of dimethyl sulfoxide (DMSO, Tedia, Fairfield, OH, USA). Next, we added 300 mg of CNTCOOH or CNTNH_2_ to the drug solution and stirred for 24 h at 300 rpm (multi-position stirrer, DAIHAN Scientific, Seoul, South Korea) at RT. To collect the loaded CNTs, we separated the solution by centrifugation and washed it twice with double-distilled water. The resulting material was oven-dried for 12 h at 50 °C and labeled as CNTCOOHDGN, CNTCOOHFUA, CNTNH_2_DGN, or CNTNH_2_FUA as appropriate.

For dual loading, we used CNTCOOHFUA and CNTNH_2_FUA as the starting materials. CNTCOOHFUA and CNTNH_2_FUA were resuspended in DGN (150 mg/15 mL organic solvent 1/1/1 DMSO/acetone/methanol) and stirred at 270 rpm (DAIHAN Scientific, Seoul, South Korea) at RT for 24 h. The same steps as described above for DGN or FUA loading were followed. The dried, loaded materials were labeled as CNTCOOHFUADGN and CNTNH_2_FUADGN.

### 2.3 Coating with Polymers: Chitosan-Conjugated Fluorescent Dye and Stearic Acid

The reason for coating with a polymer was that cancer cells preferably internalize molecules coated with sugar, acids, and antibodies.^34^ Therefore, the efficiency of drug delivery was increased due to coating. Further, the coating controls the release kinetics of the pro-drug. We used chitosan and a stearic acid–chitosan mixture, both conjugated with a fluorescent dye. The selected dye was fluorescein isothiocyanate (FI). Chitosan was selected because it is used in DDS with controlled drug release.^35^ Chitosan conjugated with fluorescent dye was called CSFI. The stearic acid was selected because it enhances cellular uptake and membrane transport.^36,37^ Stearic acid, chitosan, and Fi combination was called CSFISA. To decrease the number of investigated samples, coating was performed only for double-loaded samples: FUA and DGN.

Thus, the following materials were prepared: CNTCOOHFUADGNFUA@CSFI and CNTNH_2_FUADGN@CSFI; CNTCOOHFUADGNFUA@CSFISA and CNTNH_2_FUADGNFUA@CSFISA.

#### 2.3.1 Preparation of Chitosan-Conjugated Fluorescent Dye

Conjugation of FI with Chitosan was performed according to Mi et al.,^38^ with some modifications. We dissolved 27 mg of FI (Acros Organics, Geel, Belgium) in 40 mL of methanol (Fisher Scientific, Loughborough, UK) and added it to 40 mL of 1% chitosan (MW: 100,000–300,000, Acros Organics, Geel, Belgium) solution (in 0.1% acetic acid). The mixture solution was stirred in the dark at RT for 24 h, then centrifuged for 10 min, and then washed with double-distilled water until no green fluorescence was detected. The product (CSFI) was re-suspended in double-distilled water and kept at 5 °C until further use.

#### 2.3.2 Preparation of Chitosan–FI–Stearic Acid

To prepare this formulation, activation of the carboxylic acid groups of stearic acid was needed. It was achieved as described in our previous study.^39^ We dissolved 284 mg of stearic acid (MW: 284.48, Acros Organics, Geel, Belgium), 206 mg of 1-(3-(dimethylamino)propyl)-3-ethylcarbodiimide hydrochloride (EDC; Acros Organics, Geel, Belgium), 140 mg of *N*-hydroxysuccinimide (NHS; Acros Organics, Geel, Belgium), and 0.250 mL of triethanol amine (TEA; Molekula GmbH, Munich, Germany) in 20 mL of DMSO in a beaker. We stirred the mixture for 2 h at 80 °C and another 24 h at RT. In addition, we slowly added the activated solution containing stearic acid to the CSFI solution, stirred for 10 h at 60 °C, and kept it at RT for another 24 h. The resulting solution (CSFISA) was stored at −20 °C until further use.

#### 2.3.3 Coating the Drug-Loaded Formulation

To coat the drug-loaded CNTs with CSFI or CSFISA, we followed our previous methods.^39−41^ 300 mg portion of CNTCOOHFUADGN or CNTNH_2_FUADGN was dispersed into 10 mL of CSFI or CSFISA solution before stirring at 250 rpm at RT for 48 h. The coated materials were collected by centrifugation, washed with both ethanol and double-distilled water in succession, and then oven-dried for 12 h at 50 °C. As a result, 4 types of samples were produced.

Table 1 lists the produced samples. Table 2 lists the preparation conditions.

**Table 1:** List of the Produced Samples^a^

no.	sample name	functionalization	drug loaded	coating
	FUA			
	DGN			
	CNTCOOH			
	CNTNH_2_			
F1	CNTCOOHDGN	–COOH	DGN	
F2	CNTCOOHFUA	–COOH	FUA	
F3	CNTNH_2_DGN	–NH_2_	DGN	
F4	CNTNH_2_FUA	–NH_2_	FUA	
F5	CNTCOOHFUADGN	–COOH	DGN&FUA	
F6	CNTNH_2_FUADGN	–NH_2_	FUA	
F7	CNTCOOHFUADGNFUA@CSFI	–COOH	DGN&FUA	CSFI
F8	CNTCOOHFUADGNFUA@CSFISA	–COOH	DGN&FUA	CS&FI&SA
F9	CNTNH_2_FUADGN@CSFI	–NH_2_	DGN&FUA	CSFI
F10	CNTNH_2_FUADGN@CSFISA	–NH_2_	DGN&FUA	CS&FI&SA

a These samples were planned to be tested for cellular uptake only based on the fluorescence properties of FI dye (fluorescein isothiocyanate). Thus, they were not tested in other studies.

**Table 2:** Preparation Conditions and Physicochemical Properties of the Designed Nanoformulations for DGN and/or FUA

formula	preparation conditions			TWL and PCC (% wt)^a^	SSA (m^2^/g)^b^	total pore volume^c^ (cm^3^/g)
	drug/CNT ratio	volume/solvent	temperature/stirring speed			
CNTCOOH				15.6	233.5	0.72
CNTNH_2_				17.6	151.7	0.856
F1: CNTCOOHDGN	1/3	10 mL/DMSO	RT(24 h)/300 rpm	TWL **= 31.8**	83.3	0.49
				PCC		
				CNT = 15.6		
				DGN = 16.2		
F2: CNTCOOHFUA	1/3	10 mL/DMSO	RT(24 h)/300 rpm	TWL **= 37.7**	146.4	0.69
				PCC		
				CNT = 15.6		
				FUA = 22.1		
F3: CNTNH_2_DGN	1/3	10 mL/DMSO	RT(24 h)/300 rpm	TWL **= 34.1**	75.5	0.55
				PCC		
				CNT = 17.6		
				DGN = 16.5		
F4: CNTNH_2_FUA	1/3	10 mL/DMSO	RT (24 h)/300 rpm	TWL **= 18.4**	117.1	0.65
				PCC		
				CNT = 17.6		
				FUA = 0.8		
F5: CNTCOOHFUADGN	FUA-loaded CNTs (150 mg)/15 mL/1/1/1 (DMSO/acetone:methanol)/RT (24 h)/270 rpm			TWL **= 38.3**	71.5	0.52
				PCC		
				CNT = 15.6		
				FUADGN = 22.7		
F6: CNTNH_2_FUADGN	FUA-loaded NPs (150 mg)/15 mL/1/1/1 (DMSO/acetone/methanol)/RT (24 h)/270 rpm			TWL **= 35.7**	80.1	0.54
				PCC		
				CNT = 17.6		
				FUADGN = 18.1		
F8: CNTCOOHFUADGN@CSFISA	CNTCOOHFUADGN (300 mg)/10 mL/CS–FITC–SA/RT (48 h)/250 rpm			TWL **= 57.4**	44.0	0.38
				PCC		
				CNT = 15.6		
				FUADGN = 22.7		
				CSFISA = 19.1		
F10: CNTNH_2_FUADGN@CSFISA	CNTNH_2_FUADGN (300 mg)/10 mL/CS–FITC–SA/RT (48 h)/250 rpm			TWL **= 56.9**	40.4	0.36
				PCC		
				CNT = 17.6		
				FUADGN = 18.1		
				CSFISA = 21.2		

a Data obtained from TG analysis.

b SSA measured from BET surface area measurements.

c Total pore volume ∼0.989 *P*/*P*_0_. CNT, carbon nanotube; TWL, total weight loss; PCC, potential content of individual component; wt, weight; RT, room temperature.

### 2.4 Characterization Techniques

Images of the nanostructures were taken by means of field emission scanning electron microscopy (Zeiss Ultra Plus FE-SEM). The operating voltage was 2 kV in the lens operating mode. The crystallographic structure was analyzed by powder X-ray diffraction (XRD) using CuKα radiation with 2θ (5–100°) (Panalytical, model X’Pert PRO). Surface functional groups were measured by Fourier transform infrared (FTIR) spectroscopy using a Bruker (Billerica, MA, USA) Tensor 27 IR instrument implemented with attenuated total reflectance (Bruker Platinum ATR-Einheit A 255). We analyzed the thermal properties by simultaneous thermal analysis (STA) on a 449 F1 Jupiter instrument (NETZSCH-Feinmahltechnik GmbH, Germany). We also performed thermogravimetry (TG) and differential scanning calorimetry (DSC) measurements. For that purpose, each sample (approximately 11 mg) was heated from RT to over 1000 °C at 10 °C/min in a helium flow via the furnace chamber. Zeta potential and particle size via dynamic light scattering (DLS) were measured for each material suspended in deionized water adjusted to pH 6.8 and sonicated for about 20 min prior to the measurements. A Malvern ZetaSizer (NanoZS, Malvern Instruments Ltd., Malvern, UK) system was used. The samples were dispersed in double-distilled water adjusted to pH 6.5 and sonicated for 20–30 min before the measurements. The pH in the aqueous solution was adjusted to 6.8 to imitate the tumor environment.

### 2.5 Determination of Entrapment Efficiency and Total Drug Content

We determined the drug entrapment efficiency (EE) for all prepared nanoformulations by dissolving ∼10 mg of each nanoformulation in 10 mL of ethanol with continuous stirring for 2 h, followed by 30 min centrifugation through a high-speed cooling centrifuge at 25,000 rpm/5 °C (Sigma 3–30 KS, Sigma Laborzentrifugen GmbH, Osterode am Harz, Germany). We measured the absorbance of the supernatant at 390 nm by UV–vis spectrophotometry (Shimadzu UV spectrophotometer, 240 j/PC, Japan). The EE % was calculated as the ratio of encapsulated drug (determined by subtracting free DGN or FUA measured in the supernatant from the initial theoretically calculated amount of DGN or FUA) to the initial theoretically calculated amount of DGN or FUA (eq 1):
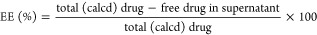
1

To quantify the total loading content (TLC) and total drug capacity (LC), approximately 10 mg of each nanoformulation was dissolved in 10 mL of ethanol with continuous high-speed stirring for 4 h. A syringe filter was used to filter the solution through a 0.2 μm filter, ensuring no CNTs were present prior to measurement of absorbance at 331 and 295 nm for DGN and FUA, respectively. LC and TLC were calculated based on eqs 2 and 3
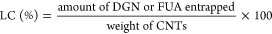
2
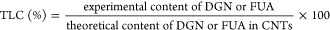
3

### 2.6 In Vitro Release Studies

The in vitro release studies were performed as described in refs (40) and (42). Phosphate buffered saline (PBS)(pH 6.8) was employed as the release medium with the addition of 10 or 20 mM glutathione (GSH), defined as low and high GSH, respectively. Five milligrams of each nanoformulation was placed in a dialysis bag (cellulose, MWCO 12,000 g/mol, Sigma-Aldrich CHEMIE GmbH, Sternheim, Germany) containing 3 mL of adjusted release medium. Both ends of the bag were tightly closed before it was immersed in 50 mL of the release medium in a capped glass bottle. The bottles were placed in an incubator (GFL 3032, Gesellschaft fur Labortechnik mbH, Burgwedel, Germany) shaking at 150 rpm at 37 °C for 72 h. At predetermined time points, 2 mL of the release medium was sampled and replaced with the same volume of the fresh medium. To measure DGN and FUA content, the removed samples were centrifuged and analyzed at 331 and 295 nm using a UV–vis spectrophotometer. The mean cumulative release of DGN or FUA at each time point was calculated from three measurements. The cumulative release data were fitted by linear or nonlinear regression using KineDS3 software (Jagiellonian University, Krakow, Poland) to determine the release kinetic model.

### 2.7 Cell Cultures

Normal human skin fibroblast (HSF) cells and three cancer cell lines from the American Type Cell Culture (ATCC) were purchased from Nawah Scientific, Cairo, Egypt: HepG2 hepatocellular carcinoma cells, MCF-7 breast adenocarcinoma cells, and A549 non-small-cell lung cancer cells. All cell lines were cultured in RPMI 1640 medium (pH > 7.2) containing 20 mM HEPES, l-glutamine, and phenol red (Sigma-Aldrich, St. Louis, MO, USA) and maintained at 37 °C in a humidified 5% CO_2_ atmosphere.

### 2.8 In Vitro Cytotoxicity Assessment

To assess anticancer effects, we used the 3-4,5-dimethylthiazol-Z-yl-2,5-diphenyltetrazolium bromide (MTT) assay according to the manufacturer’s protocol (Sigma-Aldrich, St. Louis, MO, USA). Initially, cells were cultured in 96-well flat-bottom tissue culture plates at 10^4^ cells/well and allowed to adhere and grow at 37 °C for 24 h in a humidified 5% CO_2_ atmosphere. After 24 h, we removed the medium and replaced it with fresh RPMI 1640 medium containing 100 μL of the tested material, nanoformulation, or free natural pro-drug suspended in PBS. Each nanoformulation was sonicated for 5 min, allowing the dispersion of nanoparticles in solution. To test the cytotoxicity of CNTCOOH and CNTNH_2_ nanocarriers, normal cells were treated with 100 μL of 200, 400, 800, and 1000 μg/mL and incubated for 48 h. For free DGN and FUA, cells were treated at 0.25, 0.75, 1.5, and 2 μM; in the case of nanoformulations, cells were treated with an equivalent amount of each nanoformulation to free DGN and/or FUA, and the cells were incubated for 48 h. As a control, cells were incubated with PBS. At the end of the incubation period, we removed the media and placed fresh RPMI 1640 medium containing 10 μL of MTT solution (5 mg/mL in PBS) in each well. We incubated the plates for another 4 h at 37 °C in a 5% CO_2_ atmosphere. Thereafter, we aspirated the medium containing MTT solution and added 50 μL of DMSO (Al Nasr Company, Cairo, Egypt) to each well, allowing the dissolution of MTT formazan crystals. We kept the plates at 37 °C for 30 min to ensure complete solubilization of the crystals, resulting in a purple color. Finally, for each sample, we measured the absorbance at 540 nm using a microplate reader (BMG Labtech, Ortenberg, Germany). The results were expressed as the mean cell viability % ± standard deviation (SD) and compared to the control.

### 2.9 Cellular Morphology

The cellular morphology of A549 lung cancer cells and cancer cell lines was checked using an inverted microscope (Nikon, TMS-F, New York, USA).

### 2.10 Flow Cytometry Study

The experimental methodology is described in the Supporting Information.

### 2.11 Measurement of lncRNA and miRNA Expression Level

All measurements were taken on HepG2 and HSF-normal cells. The HepG2 cells were selected because the investigated nanoformulations were more effective on HepG2 than cancer cells A549 and MCF7.

Total RNA extraction with conserved lncRNAs was achieved using the miRNeasy extraction kit (Qiagen, Valencia CA, USA), following the manufacturer’s guidelines. The control cells were treated or not with free natural agents and nanoformulations at 0.25 μM (low concentration) and 1.75 μM (high concentration) for 24 and 48 h. RNA quantitation and purity were estimated using an ND-1000 spectrophotometer (NanoDrop Technologies, Inc., Wilmington, DE, USA). We carried out reverse transcription using the RT2 first-strand kit, as recommended by the manufacturer (Qiagen, Germantown, MD, USA).

The HOTTIP, HOTAIR, HULC, CCAT2, and H19 lncRNA levels were determined using customized primers and the Maxima SYBR Green PCR kit, as directed by the manufacturer (Thermo). We used glyceraldehyde 3-phosphate dehydrogenase (GAPDH) as a control.

Real-time PCR was performed in the Rotor-gene Q System (ROTOR-Gene Q, SN R1211164, Qiagen, Hilden, Germany) under the following conditions: 95 °C for 10 min, with 45 cycles comprising 95 °C for 15 s and 60 °C for 60 s. We used the Δ−Δ CT (2^–ΔΔCt^) method to estimate the fold change in lncRNAs. The number of quantitative PCR cycles needed for the signal (i.e., fluorescence) to reach a certain threshold is called the cycle threshold (Ct). The ΔCt value is the difference between the Ct of the control (in this case, GAPDH) and the Ct of lncRNAs. The ΔΔCt value was obtained by subtracting the control ΔCt values from the ΔCt values of the samples. For the control, ΔΔCt = 0 and 20 equals 1. The miRBase accession numbers (Gene Globe IDs) were LPH15377A for HOTTIP, LPH07360A for HOTAIR, LPH01147A for H-19, LPH17802A for HULC, and LPH31725A for GAPDH. Primers were: CCAT2 (forward), 5′-CCACATCGCTCAGACACCAT-3′; and (reverse), 5′-ACCAGGCGCCCAATACG-3′.

We used the miScript SYBR Green PCR kit to quantify the expression of miRNAs 21, 92, 145, and 181a according to the manufacturer’s instructions. We used the following cycling conditions: 94 °C for 10 min, with 40 cycles comprising 94 °C for 15 s, 55 °C for 30 s, and 70 °C for 30 s. SNORD 68 (catalog no. MS00033712) was used as an internal control. The miRBase accession numbers (Gene Globe IDs) were MS00009079 (miR-21), MS00006594 (miR-92), MS00003528 (mir-145), and MS00008827 (mir-181a).

### 2.12 Statistical Analysis

The results of the biological evaluations are expressed as the mean values ± SD. Significant differences were determined by a *t*-test (for cytotoxicity data) and a one-way analysis of variance (ANOVA) (for biological evaluations) in GraphPad PRISM (Version 8.0.1, GraphPad Software, San Diego, CA, USA). We set *p* < 0.05 as significant.

### 2.13 Schematic Presentation of the Research Plan

**Scheme 1:**
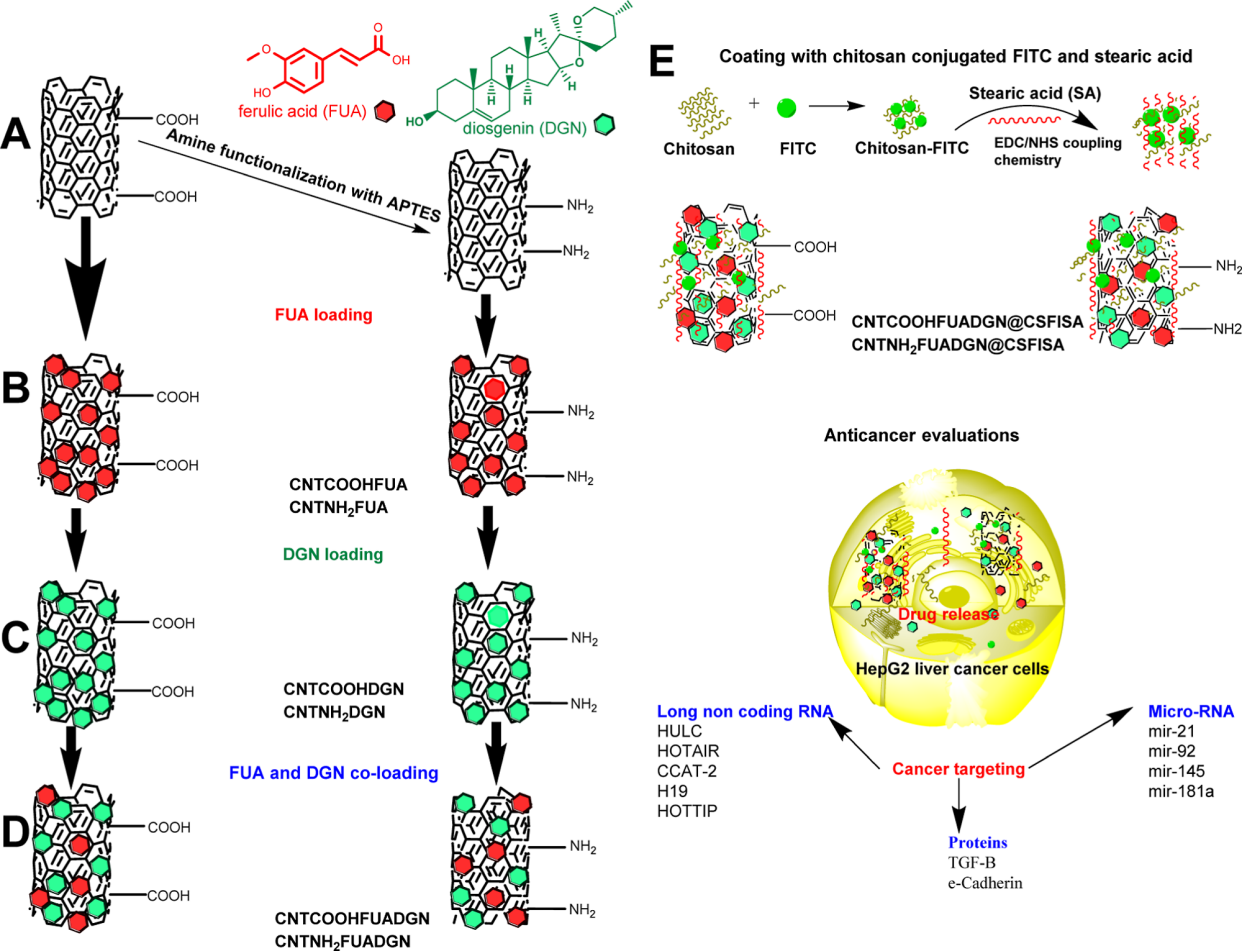
Preparation of the Delivery System for Single and Dual Loading of Natural Agents to Obtain Different Anticancer Nanoformulations for Evaluation (A–D) Surface Modification of CNTNH_2_ through the Reaction of CNTCOOH with APTES Silane Molecules, Enabling –NH_2_ on the Surface of Nanotubes (A), FUA Loading of CNTs to Obtain CNTCOOHFUA (F2) and CNTNH_2_FUA (F3) (B), DGN Loading of CNTs to Obtain CNTCOOHDGN (F1) and CNTNH_2_DGN (F4) (C), Dual Loading of CNTs to Obtain CNTCOOHFUADGN (F5) and CNTNH_2_FUADGN (F6), Where the FUA-Loaded Nanotubes Were Used to Subsequently Load DGN (D). Coating with Chitosan–Stearic Acid to Obtain CNTCOOHFUADGN@CSFISA (F8) and CNTNH_2_FUADGN@CSFISA (F10) (E). The Dual-Loaded Nanotubes Were Used for mRNA Tests (Bottom, Right).

Scheme 1 schematically summarizes the research plan.

## 3 Results and Discussion

### 3.1 Structural Studies

#### 3.1.1 Morphological Observations

**Figure 1:**
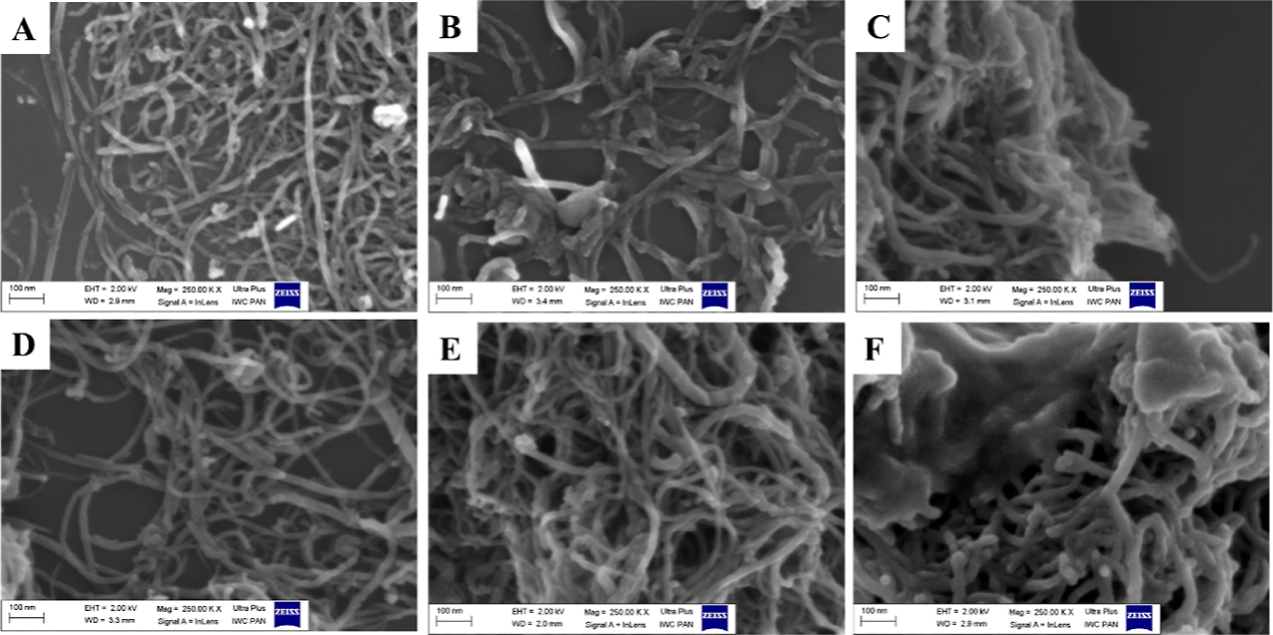
FE-SEM images taken for different preparation steps to detect morphological changes. (A) CNTCOOH, (B) F5—CNTCOOHFUADGN, (C) F8—CNTCOOHFUADGN@CSFISA, (D) CNTNH_2_, (E) F6—CNTNH_2_FUADGN, and (F) F10—CNTNH_2_FUADGN@CSFISA. The images show no changes before and after surface modification and dual loading of DGN and FUA, but there was an obvious change after coating with the chitosan–stearic acid complex (compare C,F). This observation indicates the attachment of chitosan/stearic acid on the nanotube surface.

Figure 1 shows the FE-SEM images of CNTs and nanoformulations. No differences were seen in the morphologies of CNTCOOH (Figure 1A), CNTCOOHFUADGN (Figure 1B), CNTNH_2_ (Figure 1D), and CNTNH_2_FUADGN (Figure 1E). Characteristic images of the entanglements of MWCNTs are seen. Figure 1C,F shows a coating on the surface of the samples, as expected for the coated CNTCOOHFUADGN@CSFISA and CNTNH_2_FUADGN@CSFISA, respectively.

#### 3.1.2 Surface Area Measurement

To determine the change in the CNT structure caused by drug loading, we measured the SSA and total pore volume for the materials. The results are listed in Table 2. The SSA and total pore volumes of the materials decreased with surface modification, drug loading, and polymer coating. For example, the surface area decreased from 233.5 m^2^/g for CNTCOOH to 146.4 m^2^/g after loading with FUA (CNTCOOHFUA), 83.3 m^2^/g after loading with DGN (CNTCOOHDGN), 71.6 m^2^/g after dual loading (CNTCOOHFUADGN), and 44.0 m^2^/g after coating (CNTCOOHFUADGN@CSFISA). This is an expected sequence for an increase of the CNT diameter due to drug loading and coating. It is seen that FUA loading causes less changes than DGN loading. A possible reason for the difference between CNTs loaded with DGN and FUA could be caused by a difference in the loading capacity and/or molecular mass of the agents. The total pore volume decreased from 0.72 cm^3^/g for CNTCOOH to 0.49, 0.69, 0.52, and 0.38 cm^3^/g for CNTCOOHDGN, CNTCOOHFUA, CNTCOOHFUADGN, and CNTCOOHFUADGN@CSFISA, respectively, in line with SSA changes.

#### 3.1.3 Measurement of CNT’s Size and Zeta Potential

The size distribution of the CNTs suspended in water is shown in Figure S1 and Table S1 in the Supporting Information. For the interpretation of these results, it is to consider that the DLS method will detect size of particles in the form of entangled MWCNTs or their agglomerates. It was observed that polymer coating results in a significant increase in mean size. The samples without polymer coating display a mean size in the range 235–585 nm, while the coated ones are in the range 792–1191 nm. This result may be caused by agglomeration. These results are in line with previous data for CNT loaded with drugs.^43^

All of the materials showed negative surface charges (Figure S2 and Table S1, Supporting Information). High negative zeta potentials cause electrostatic repulsion between negatively charged clusters, enhancing the stability of nanoformulations in aqueous solutions.^44^ This is advantageous as stable suspensions of DDS are needed to deliver to the cells the drug loaded in them.

#### 3.1.4 Characterization by Means of XRD

**Figure 2:**
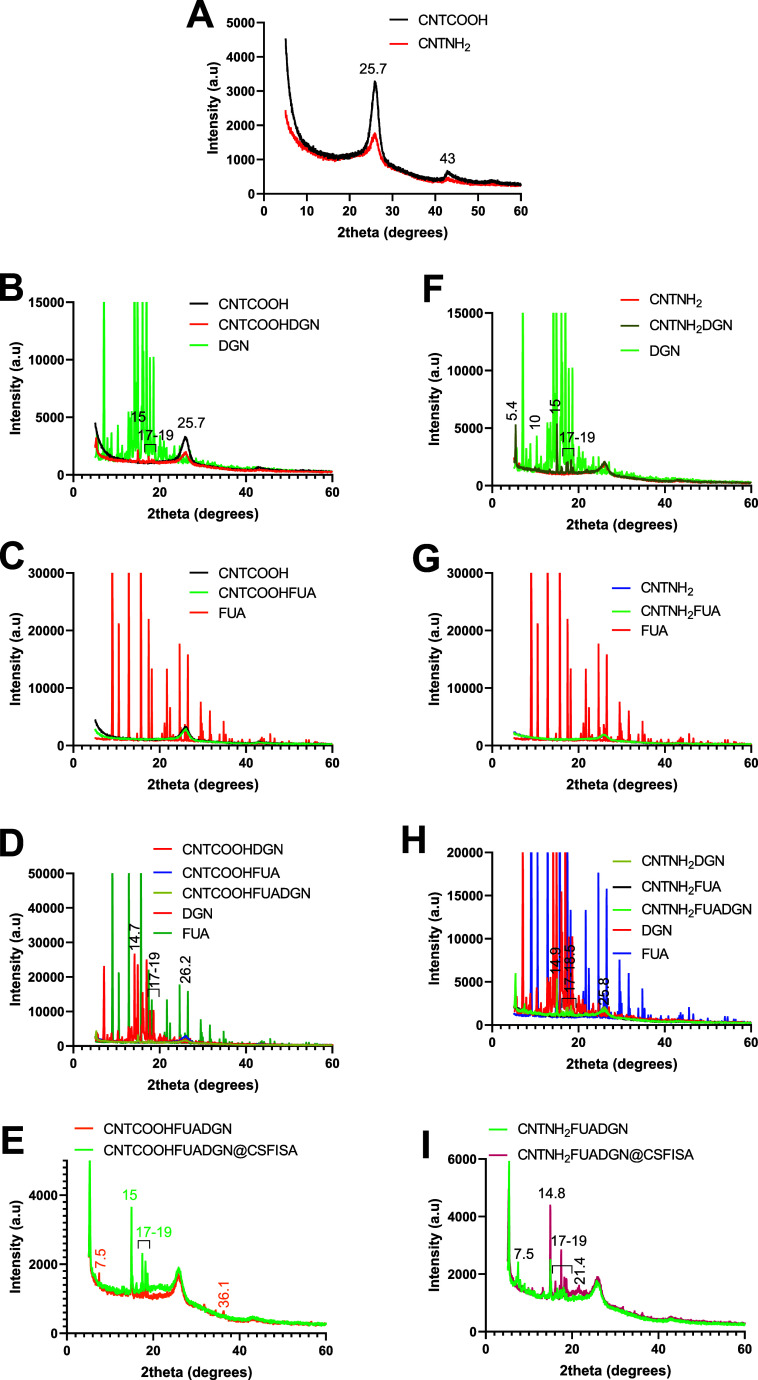
XRD patterns of CNTs before and after surface modification, natural agent loading, and coating with a chitosan–stearic acid complex. (A) CNTs before (CNTCOOH) and after surface modification with amino groups (CNTNH_2_). (B–E) Nanoformulations designed using CNTCOOH-based nanotubes. (F,I) Nanoformulations constructed using CNTNH_2_-based nanotubes. (B,F) Compare the nanoformulation before and after DGN loading with free DGN. Loading caused DGN peaks to be seen. (C,G) Compare the nanoformulation before and after FUA with free FUA. (D,H) Compare the nanoformulations containing single-loaded DGN or FUA and dual loading with free DGN and FUA. (E,I) Compare the nanoformulations before and after chitosan–stearic acid complex attachment.

As shown in Figure 2A, the XRD patterns of both CNTCOOH and CNTNH_2_ presented two signals at 2θ = 25.7° and 2θ = 43.0°. These peaks indexed as C(002) and (100) reflect the hexagonal structure of CNTs. The high intensity and sharpness of peak C(002) indicate the multi-walled morphology of the CNTs. Surface modification (CNTNH_2_) resulted in a decreasing intensity, which may be the result of functionalization with –NH_2_ due to the APTES molecules attaching to the nanotube surface. New peaks in the 2θ region between ∼15 and 19° appeared when DGN was loaded (CNTCOOHDGN, CNTNH_2_DGN, CNTCOOHFUADGN, and CNTNH_2_FUADGN), ascribed to free DGN (Figure 2B,F,D,H). The intensity of the main typical peak of CNTs at 2θ = 25.7° decreased, as indicated in CNTCOOHDGN, due to the attachment of DGN molecules in the nanotubes. This indicates an interaction of the pro-drug molecules with the CNTs and changes in CNT structure. The new peaks demonstrate that DGN molecules attached to the CNT surface have a crystalline structure. A small modification of the XRD pattern is seen for FUA loading (CNTCOOHFUA and CNTNH_2_FUA) (Figure 2C,G). No FUA peaks were detected, which indicates that FUA molecules form an amorphous or very thin layer.

The nanoformulations before and after coating with chitosan–stearic acid are compared in Figure 2E,I. After coating with both polymers, new peaks appeared at 2θ = 15 and 17 to 19° for CNTCOOHFUADGN@CSFISA, and peaks at 2θ = 7.5 and 36.1° for CNTCOOHFUADGN disappeared. Similarly, several new peaks appeared at 2θ = 14.8, 17–19, and 21.4° for CNTNH_2_FUADGN@CSFISA, but a peak at 7.5° disappeared compared to CNTNH_2_FUADGN. Disappearing and appearing peaks after and before coating could indicate interactions between the loaded drugs and the coating layer.

#### 3.1.5 Thermal Characterization

The results of the TG/DSC investigations are presented in Figure S3 in the Supporting Information and Table 2. The total weight loss (TWL %) and the potential content of individual components (PCC %), determined as the difference in the results of the respective experiments, were determined. The weight loss for nonloaded CNTs is in the range of 16–18%. Loading with DGN and/or FUA results in weight loss in the range of 32–38%, depending on the type of CNT used. An exception is the F4—CNTNH_2_FUA, where 18 wt % weight loss was observed. For polymer coating, the weight loss was in the range of 57 wt %, indicating a successful coating.

#### 3.1.6 FTIR Measurements

**Figure 3:**
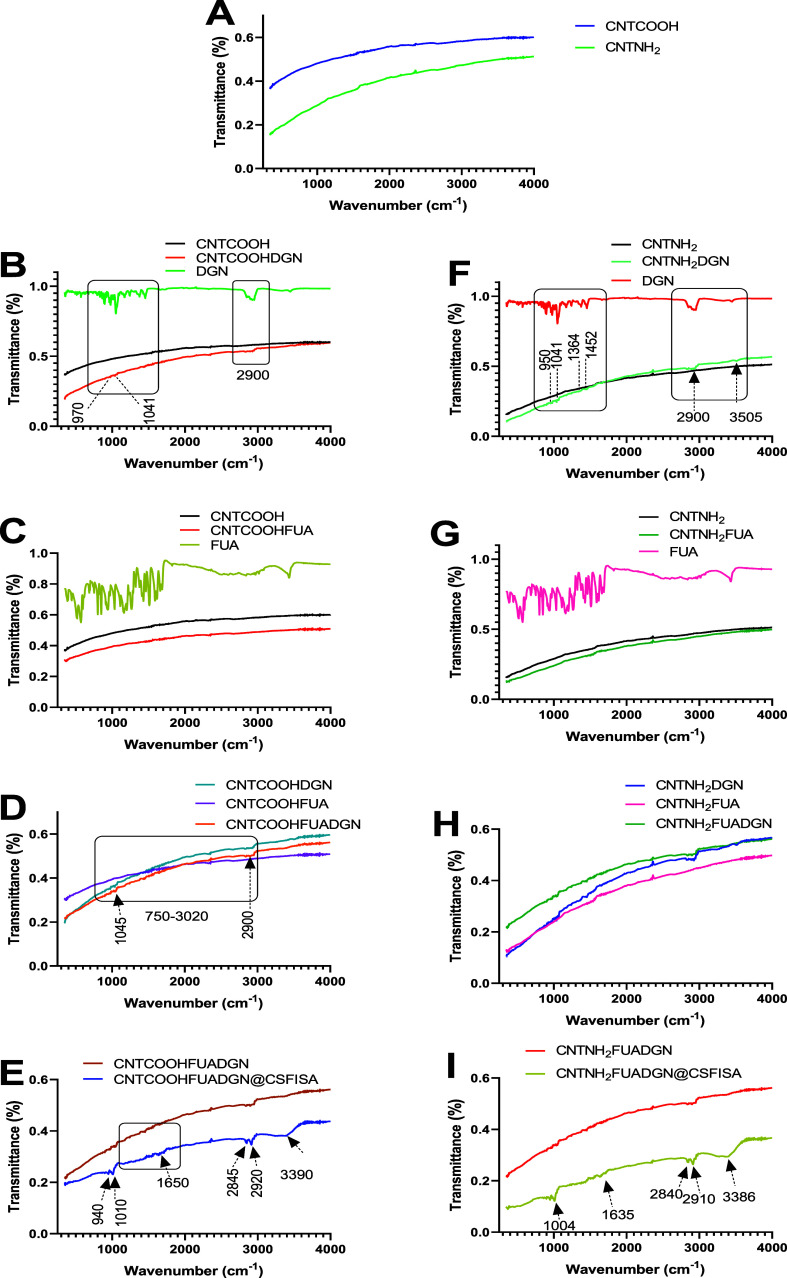
FTIR spectra of CNTs before and after surface modification, natural agent loading, and coating with a chitosan–stearic acid complex. (A) CNTCOOH and CNTNH_2_. (B–E) CNTCOOH-nanotubes. (F,I) CNTNH_2_-nanotubes. (B,F) Nanoformulations before and after DGN loading with free DGN. (C,G) Compare the nanoformulation before and after FUA loading with free FUA. (D,H) Compare the nanoformulations loaded with DGN or FUA and dual loading with free DGN and FUA. (E,I) Compare the nanoformulations before and after chitosan–stearic acid coating.

FTIR measurements were carried out to identify surface functional groups (Figure 3). Reference spectra of FUA and DGN were typical for these materials (Supporting Information). After loading CNTs with DGN, additional very weak peaks were seen at 970 and 1049 cm^–1^ (Figure 3B,F). Bands assigned to DGN were shifted compared to the FTIR spectra of the pure substance. New bands detected in the CNTCOOHDGN and CNTNH_2_DGN spectra included 3514, 2946, 1446, 1171, 1067, and 899 cm^–1^ (Figure 3B,F), indicating interactions between DGN and the MWCNTs. For both CNTCOOHFUA and CNTNH_2_FUA, no new peaks were recorded (Figure 3C,G), even though CNTCOOHFUA showed a weight loss of about 37 wt % compared to CNTNH_2_FUA. This observation demonstrates that the FTIR signal for FUA is not related to the loading capacity into these two nanoformulations. FUA is perhaps entrapped in the CNTCOOH or CNTNH_2_ network structure, preventing its detection by FTIR. The data for CNTCOOHFUADGN and CNTNH_2_FUADGN were very similar. The signals related to DGN are seen rather than those related to FUA (Figure 3D,H).

FTIR spectra of the CNT samples before and after coating with chitosan–stearic acid confirm the effectiveness of the surface modification. For CNTCOOHFUADGN@CSFISA and CNTCNH_2_FUADGN@CSFISA, a wide band centered at 3369 cm^–1^ attributed to amine NH symmetric vibration and weak sharp peaks at 1644 cm^–1^ (amide I C=O stretching), 1556 cm^–1^ (amide II N–H bending), and 1150 and 899 cm^–1^ (saccharide structure of chitosan) confirmed the presence of chitosan.^41^ Relatively intense signals assigned to stearic acid were recorded at 2914 and 2847 cm^–1^ (asymmetric and symmetric –CH_2_– stretching vibrations, respectively) and at 1695 cm^–1^ (−COOH bond).^45^ This observation is consistent with our previous results obtained for a DDS based on mesoporous silica nanoparticles coated with thymoquinone-loaded chitosan–stearic acid polymers.^41^

#### 3.1.7 Summary of Structure Studies

The MWCNTs have been successfully functionalized with –COOH or –NH_2_. DGN loading as well as polymer coating were confirmed by an increase of particle’s size, FTIR studies, TG studies, and morphology studies. The nanoparticles dispersed in water adjusted to pH 6.8 have a size range of 270–600 nm before polymer coating and 900 to 1100 nm after polymer coating, as needed for an efficient DDS.

### 3.2 Drug Loading and Release Studies

#### 3.2.1 Drug Loading Properties

The key feature of the DDS is the drug EE. Tables 2 and 3 show that in CNTCOOHFUADGN@CSFISA EE reached 93% for DGN and 84% for FUA, respectively. A lower EE % was detected for DGN and FUA in CNTNH_2_FUADGN@CSFISA compared to CNTCOOHFUADGN@CSFISA. The EE of DGN was higher than that of FUA. EE increased with dual DGN and FUA loading compared to single loading. The total loading TLC was close to the PCC calculated based on the TG results (compare Tables 2 and 3). The exception is CNTNH_2_FUA, for which PCC was approximately 1%, while 20% was detected by the UV–vis method, as shown in Table 3. The reason for these differences between FUA loading percentages by TG and UV–vis spectrometry can be attributed to the interaction between FUA and CNTNH_2_. In general, DGN showed a slightly higher TLC than FUA, reaching 23 and 20% for DGN and FUA, respectively, in CNTNH_2_DGN and CNTNH_2_FUA. With respect to the role of surface functionalization, the loading efficiency may relate to the strong interaction of DGN and/or FUA with a sidewall and nanotube ends via π–π stacking, hydrogen bonds, and hydrophobic interactions.^46^

**Table 3:** Loading Properties of Nanoformulations Containing Single or Dual DGN and/or FUA Natural Agents Based on the UV–Vis Spectroscopy Method^a^

sample	nanoformulation	EE %	TDC %
F1	CNTCOOHDGN	68.9 ± 4.3	18.4 ± 1.0
F2	CNTCOOHFUA	61.7 ± 2.8	18 ± 1.1
F3	CNTNH_2_DGN	65.8 ± 3.7	23.2 ± 1.0
F4	CNTNH_2_FUA	60.2 ± 5.2	19.8 ± 1.2
F5	CNTCOOHFUADGN (as FUA)	72.8 ± 4.4	14.1 ± 0.8
F5	CNTCOOHFUADGN (as DGN)	76.1 ± 4.6	16.3 ± 0.4
F6	CNTNH_2_FUADGN	69.3 ± 3.0	15.2 ± 1.1
F6	CNTNH_2_FUADGN	72.4 ± 3.8	11.5 ± 1.0
F8	CNTCOOHFUADGN@CSFISA (as DGN)	93.0 ± 2.6	12.4 ± 0.9
F8	CNTCOOHFUADGN@CSFISA (as FUA)	84.2 ± 2.0	11.8 ± 0.8
F10	CNTNH_2_FUADGN@CSFISA	87.2 ± 3.0	12.7 ± 0.8
F10	CNTNH_2_FUADGN@CSFISA	79.9 ± 2.2	11.9 ± 0.7

a Values are given as the mean ± SD. EE, entrapment efficiency; TDC, total loading content; DGN, diosgenin; and FUA, ferulic acid, mean the calculated % of each in dual-loaded nanoformulation.

#### 3.2.2 In Vitro Release Kinetics

**Figure 4:**
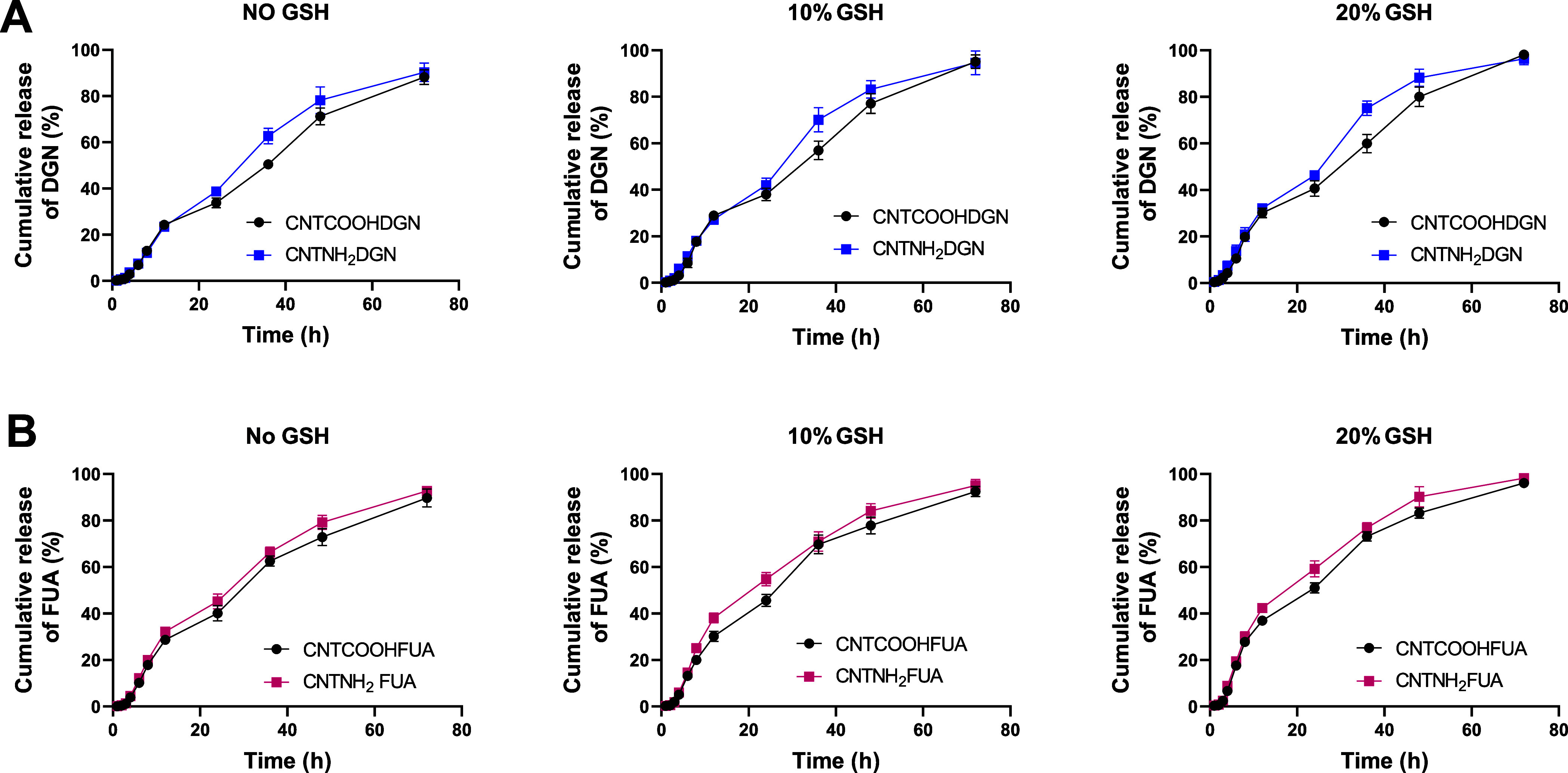
In vitro release of DGN or FUA from single-loaded nanoformulations made of CNTCOOH and CNTNH_2_ in the presence or absence of GSH. (A) DGN from nanoformulations. (B) FUA from nanoformulations. PBS was used as the medium during the experiments. Data are provided as the mean ± SD.

**Figure 5:**
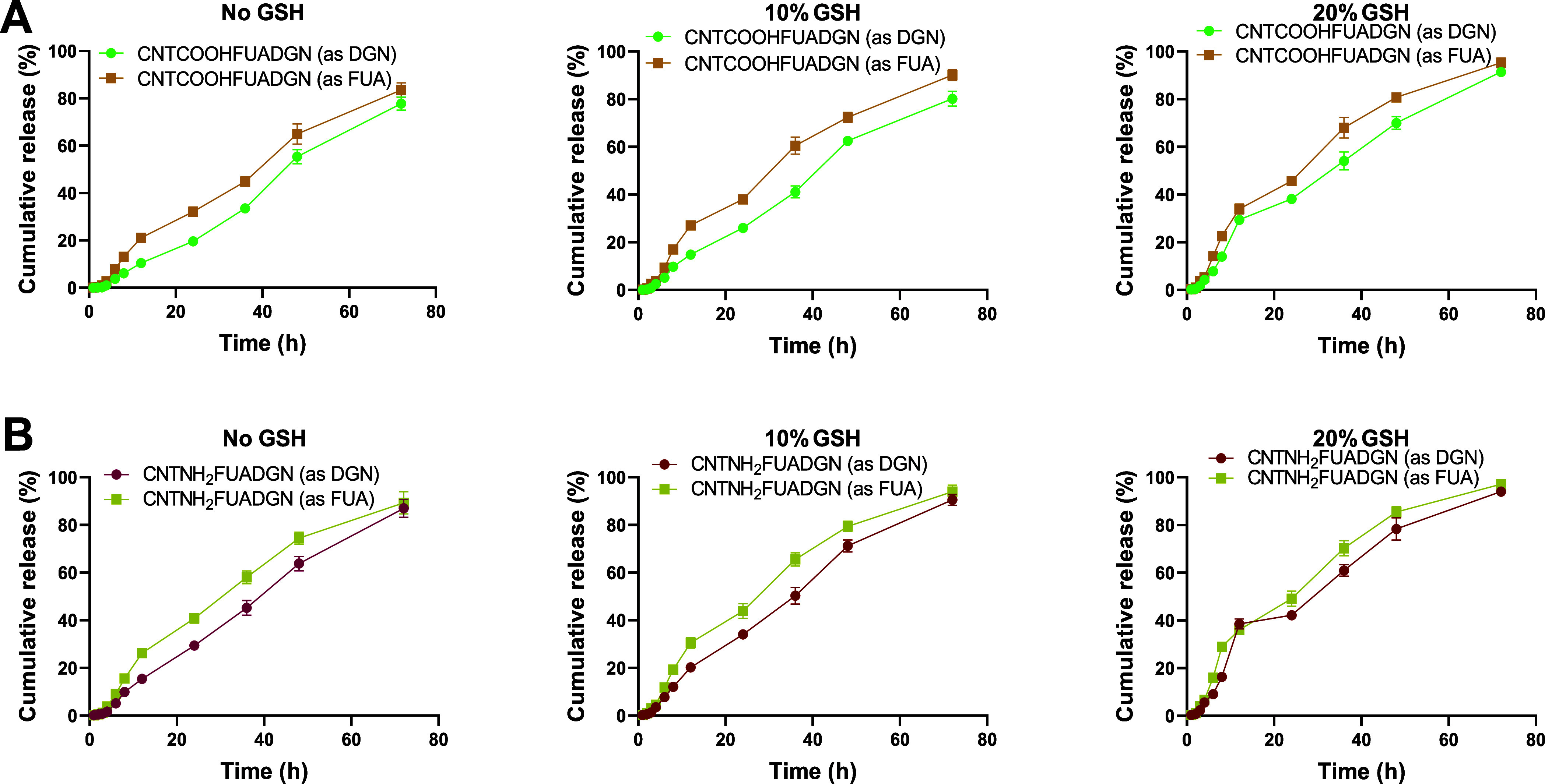
In vitro release of dual-loaded DGN and FUA from non-coated nanoformulations made of CNTCOOH and CNTNH_2_ as a function of GSH content. (A) Nanoformulation based on nonmodified CNTs and (B) nanoformulation based on amino-modified CNTs. Data are provided as the mean ± SD.

The release data values and kinetics are shown in the Supporting Information in Table S2. Figure 4A shows the DGN release from both CNTCOOHDGN and CNTNH_2_DGN in the presence of no GSH, 10% GSH, or 20% GSH and sustained release patterns. In the first stage up to about 12 h, a zero-order release effect was observed. The DGN release then gradually increased up to 72 h. Both nanoformulations had a higher cumulative release effect at a higher GSH concentration, with the highest DGN release observed for CNTNH_2_DGN (vs CNTCOOHDGN) from 24 to 48 h before the values grew close after 72 h, reaching >95%. FUA release from both nanoformulations under the same conditions exhibited the same patterns (Figure 4B), even though a higher content of DGN was loaded into CNTs. Figure 5 presents the dual delivery nanoformulations (CNTCOOHFUADGN and CNTNH_2_FUADGN), we calculated the DGN and FUA release from each nanoformulation. The release profiles demonstrated a two-stage sustained release effect, and the release changed depending on the presence of GSH. We observed a higher release of DGN and FUA from CNTNH_2_FUADGN compared to that of CNTCOOHFUADGN. Furthermore, even though FUA had a lower total loading than DGN (Table 3), it was released at higher levels than DGN. The nanoformulations loaded with dual agents released less pro-drugs than the nanoformulations loaded with a single agent. We presume that the two drugs interact with each other to decrease the release rate. For example, in the absence of GSH, CNTCOOHFUADGN released approximately 77.9% DGN and 83.5% FUA, whereas CNTCOOHDGN released >88% and CNTCOOHFUA released ∼90%. At 20% GSH, CNTCOOHFUADGN released ∼91.5% DGN and 95.5% FUA, whereas CNTCOOHDGN released ∼98.2% and CNTCOOHFUA released ∼96.1%.

**Figure 6:**
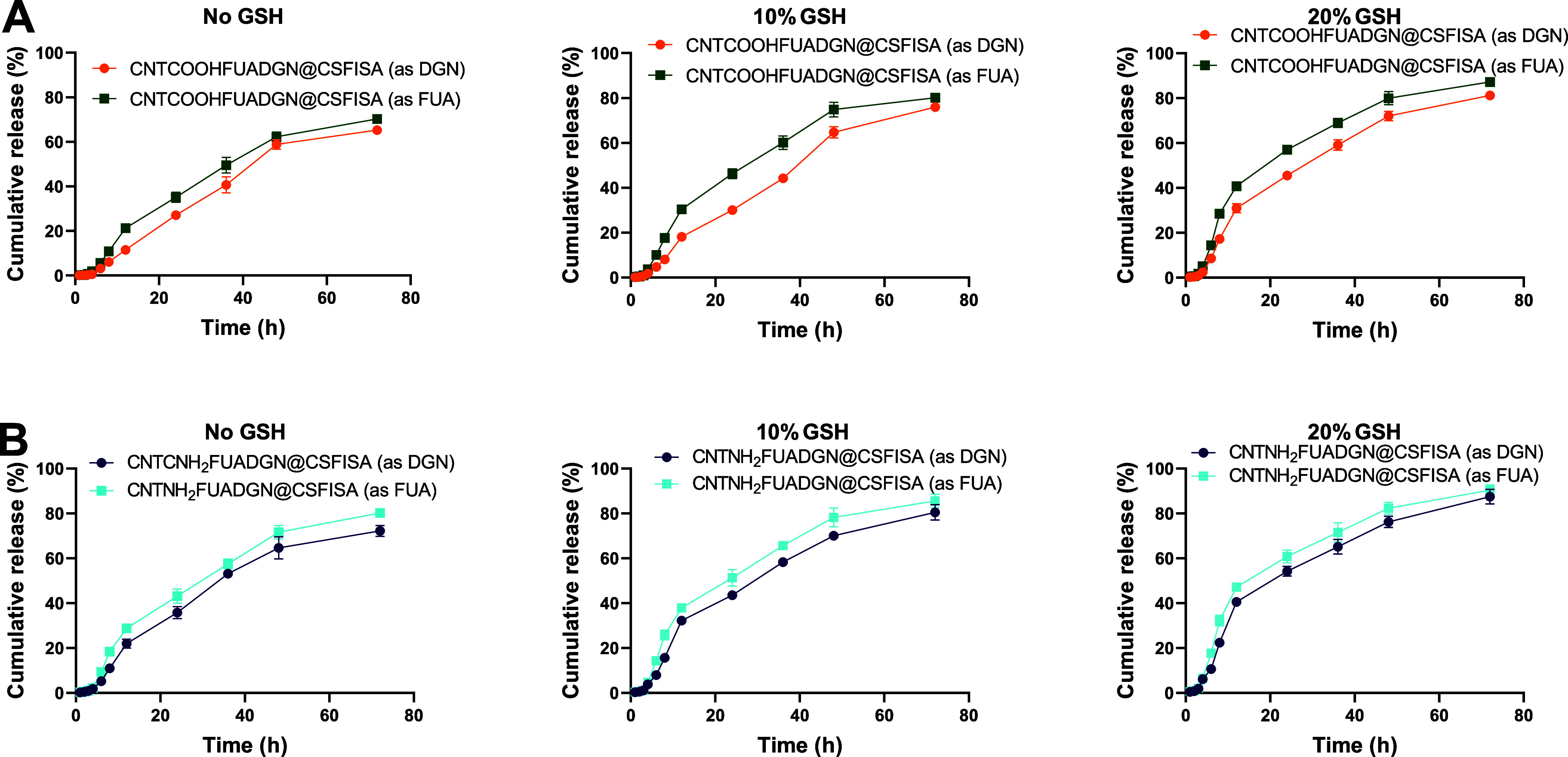
In vitro release of dual-loaded DGN and FUA from nanoformulations coated with chitosan–stearic acid for CNTCOOH and CNTNH_2_ and as a function of GSH content. (A) Nanoformulation based on nonmodified CNTs and (B) nanoformulation based on amino-modified CNTs. PBS was used as a medium during the experiments. Data are provided as the mean ± SD.

Figure 6A,B depicts the sustained release characteristics of nanoformulations with the chitosan–stearic acid polymeric coating. The release from CNTNH_2_FUADGN@CSFISA was higher than the release from CNTCOOHFUADGN@CSFISA, and FUA was released at higher levels than DGN. For example, exposing CNTNH_2_FUADGN@CSFISA to 20% GSH for 72 h released ∼87.5% DGN and ∼90.5% FUA, whereas CNTCOOHFUADGN@CSFISA released ∼81.1% and ∼87.2% as DGN and FUA, respectively. CNTCOOHFUADGN@CSFISA and CNTNH_2_FUADGN@CSFISA released lower percentages than CNTCOOHFUADGN and CNTNH_2_FUADGN (Figure 5); the maximum cumulative release was approximately 90%. The chitosan–stearic acid coating slows the release. This delay depends on the pH and GSH concentration and should be optimized to a level favorable for the tumor microenvironment. The coating effects are in line with the study by Karimi et al.,^47^ who investigated MWCNTs loaded with methotrexate and coated with polyethylene amine (PEI) and folic acid, which slowed the drug release (65%) and decreased the burst release compared to MWCNTs loaded with methotrexate alone (83% release).

The plausible reason for a higher cumulative release of FUA compared to that of DGN throughout the investigation may relate to the differences in interactions between these molecules and the aromatic surface of CNTs via π–π.^48^ It may also be associated with low free FUA content compared to high DGN content in nanoformulations, facilitating the fast release effect.

These findings are promising because this DDS with dual agents released under altering the pH/GSH is useful to target cancer cells in an acidic tumor microenvironment with high GSH levels.^49,50^

Fitting the release profile data to kinetic models showed that DGN and/or FUA releases best fit the Korsmeyer–Peppas model (Table 4). The release from nanoformulations corresponded to different kinetic models (Korsmeyer–Peppas, Zero-order, and Baker–Lonsdale), especially for nanoformulations containing both agents. The release of DGN and FUA from CNTCOOHFUADGN and DGN from CNTNH_2_FUADGN had zero-order kinetics in the absence of GSH. CNTCOOHFUADGN released DGN by zero-order kinetics in the presence of 10% GSH. In contrast, CNTNH_2_FUADGN@CSFISA released both DGN and FUA through Baker-Lonsdale kinetics in the presence of 20% GSH. It is seen that the kinetics depend on the conditions of the release media. Our results are in line with previously published papers. For example, the release of methotrexate and cyclophosphamide fits the Higuchi and Korsmeyer–Peppas kinetic models, respectively, depending on pH and temperature.^51^ Chudoba et al.^52^ reported that the best-fit release kinetics of doxorubicin from modified CNTs are due to Korsmeyer–Peppas/Ritger–Peppas kinetics (power-law model) and release controls by Fickian diffusion. Other studies have shown different kinetic models for releasing drugs from CNTs, such as the release of the natural agent silibinin from MWCNTs, which best fits pseudo-second-order kinetics.^53^ The results suggest that the release of both DGN and FUA from nonmodified or modified CNTs is largely due to Korsmeyer–Peppas kinetics and controlled by Fickian diffusion.

**Table 4:** Kinetics of the Release of DGN and/or FUA from Nanoformulations in the Presence or Absence of GSH

nanoformulation	kinetics under various conditions					
	no. GSH		GSH (10%)		GSH (20%)	
	*R*^2^	model	*R*^2^	model	*R*^2^	model
F1 CNTCOOHDGN	0.9888	Korsmeyer–Peppas	0.9902	Korsmeyer–Peppas	0.9935	Korsmeyer–Peppas
F2 CNTCOOHFUA	0.9845	Korsmeyer–Peppas	0.9892	Korsmeyer–Peppas	0.9894	Korsmeyer–Peppas
F3 CNTNH_2_DGN	0.9892	Korsmeyer–Peppas	0.9927	Korsmeyer–Peppas	0.9951	Korsmeyer–Peppas
F4 CNTNH_2_FUA	0.9878	Korsmeyer–Peppas	0.9890	Korsmeyer–Peppas	0.9912	Korsmeyer–Peppas
F5 CNTCOOHFUADGN (as DGN)	0.9907	zero-order	0.9907	zero-order	0.9911	Korsmeyer–Peppas
F5 CNTCOOHFUADGN (as FUA)	0.9849	zero-order	0.9911	Korsmeyer–Peppas	0.9933	Korsmeyer–Peppas
F6 CNTNH_2_FUADGN (as DGN)	0.9955	zero-order	0.9909	Korsmeyer–Peppas	0.9930	Korsmeyer–Peppas
F6 CNTNH_2_FUADGN (as FUA)	0.9872	Korsmeyer–Peppas	0.9920	Korsmeyer–Peppas	0.9936	Korsmeyer–Peppas
F8 CNTCOOHFUADGN@CSFISA (as DGN)	0.9782	Korsmeyer–Peppas	0.9858	Korsmeyer–Peppas	0.9874	Korsmeyer–Peppas
F8 CNTCOOHFUADGN@CSFISA (as FUA)	0.9874	Korsmeyer–Peppas	0.9882	Korsmeyer–Peppas	0.9911	Korsmeyer–Peppas
F10 CNTNH_2_FUADGN@CSFISA (as DGN)	0.9894	Korsmeyer–Peppas	0.9899	Korsmeyer–Peppas	0.9924	Baker–Lonsdale
F10 CNTNH_2_FUADGN@CSFISA (as FUA)	0.9888	Korsmeyer–Peppas	0.9894	Korsmeyer–Peppas	0.9937	Baker–Lonsdale

#### 3.2.3 Summary of Drug Loading and Release Studies

It can be seen that the prepared nanoformulations are efficient nanocarriers. EE coefficients ranging from 60 to 90% have been achieved. Dual loading has led to higher loading than single loading. CNTNH_2_-based NTs are preferred over CNTCOOH-based NTs for loading a single agent. Coating with CSFISA permitted us to achieve an EE above 80%.

### 3.3 Evaluation of Biological Activity of the CNTs and Nanoformulations

#### 3.3.1 Cytocompatibility Evaluation

**Figure 7:**
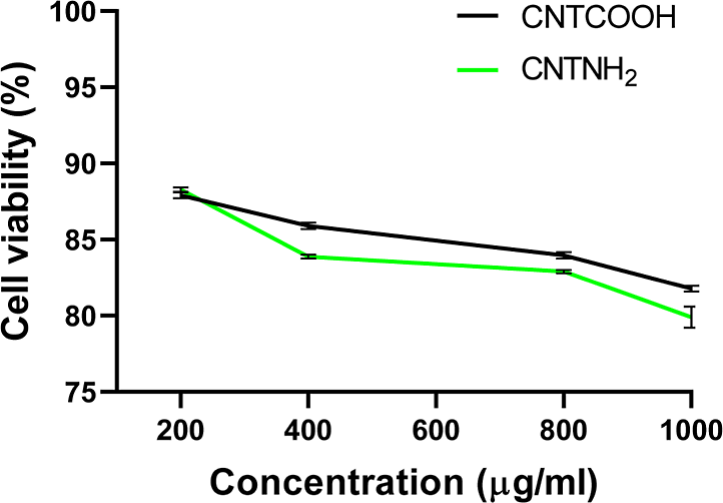
Assessment of the cytocompatibility of CNTCOOH and CNTNH_2_. Normal human skin fibroblasts (HSFs) were treated at different concentrations (μg/mL) for 48 h. Data are provided as the mean ± SD, *N* = 2 replicates. *t*-Test at significant differences among means (*P* < 0.05) [*P* value: 0.6160, *P* value summary: ns (non-significant)]. Cell viability (% of control).

Figure 7 illustrates that the cytocompatibility of both functionalized CNTs depends on the concentration used. At a high concentration (1000 μg/mL), cell viability was reduced to 81.78 ± 0.19 and 79 ± 0.69% for CNTCOOH and CNTNH_2_, respectively. No significant differences were found between the two nanocarriers. These results are consistent with previous data for CNT, CNT-modified polydopamine, and CNT-modified alginate on MC3T3-E1 and HepG2 cells, demonstrating that the cytocompatibility is concentration-dependent.^54^ The obtained results may indicate that cytocompatibility relies on the concentration of CNT used as the drug nanocarrier.^11,55^

#### 3.3.2 Anticancer Efficiency and Apoptosis Induction

**Figure 8:**
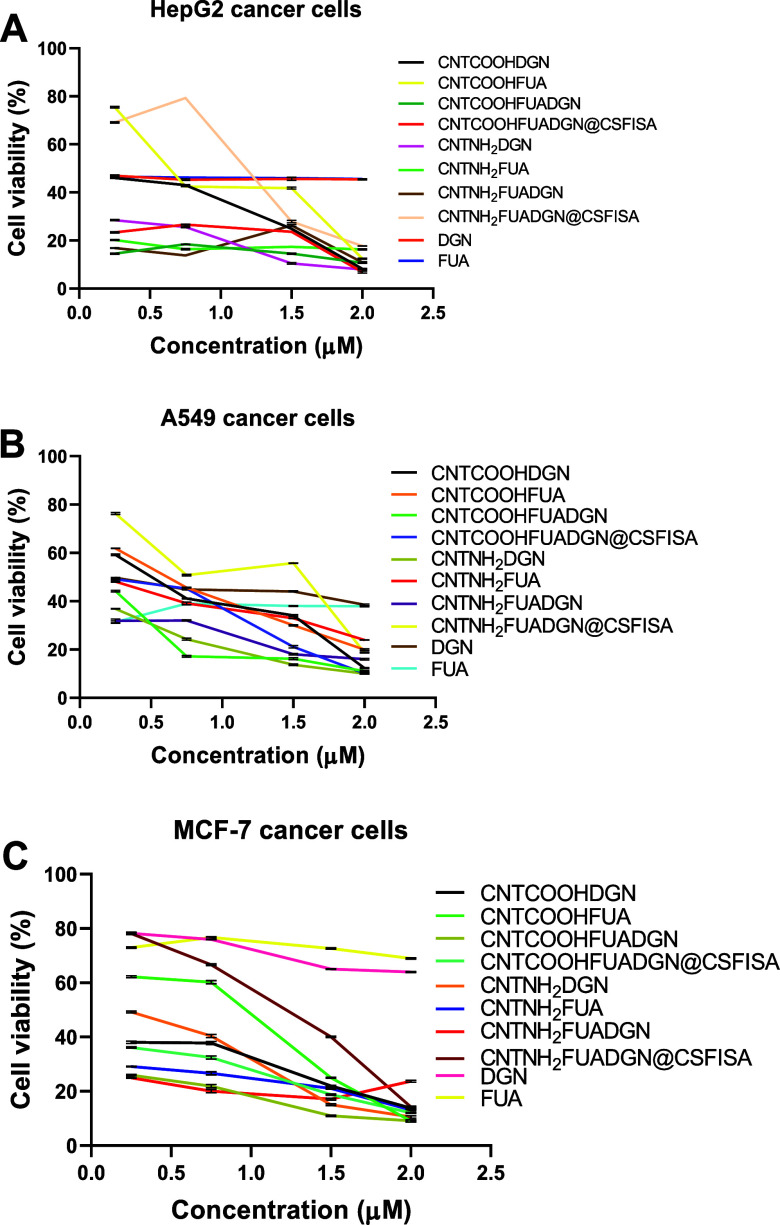
Anticancer effects of nanoformulations compared to free DGN and FUA on liver, lung, and breast cancer cell lines after 48 h exposure at different concentrations. (A) HepG2 liver cancer cells, (B) A549 lung cancer cells, and (C) MCF7 breast cancer cells. Data are provided as the mean ± SD, *N* = 2 replicates. Significant differences among means (*P* < 0.05); for HepG2 cells (*P* value: 0.003, *P* value summary: **), for A549 cells [*P* value: 0.147, *P* value summary: ns (non-significant)], and for MCF7 cells (*P* value: < 0.0001, *P* value summary: ****), cell viability (% of control).

As cell viability decreases, the anticancer effect increases (Figure 8). In the case of HepG2 liver cancer cells, Figure 8A clearly shows a concentration-dependent anticancer effect against HepG2 cells, and the cell viability declined as the concentration increased, as expected. The differences between the tested samples depend on concentrations. Compared with both free DGN and FUA, the nanoformulations exhibited a significant anticancer effect (Table 5). Free DGN and FUA had the lowest inhibition of cell viability, 45.5 ± 0.26 and 45.6 ± 0.05%, respectively. At 2 μM, all nanoformulations inhibited the viability of HepG2 cells.

**Table 5:** Shows the Ranking Effects on Cell Viability of HepG2 and A549 Cells Depending on the Nanoformulation Type

formulation	sample	viability % (HepG2) cancer cells	viability % (A549) cancer cells
F8	CNTCOOHFUADGN@CSFISA	6.7 ± 0.0	10.1 ± 0.3
F3	CNTNH_2_DGN	7.9 ± 0.3	10.0 ± 0.25
F1	CNTCOOHDGN	8.2 ± 0.2	12.3 ± 0.25
F5	CNTCOOHFUADGN	10.7 ± 0.25	11.05 ± 0.25
F6	CNTNH_2_FUADGN	10.9 ± 0.25	16.1 ± 0.3
F2	CNTCOOHFUA	12.4 ± 0.25	20.0 ± 0.3
F4	CNTNH_2_FUA	16.3 ± 0.25	23.9 ± 0.1
F10	CNTNH_2_FUADGN@CSFISA	17.7 ± 0.08	18.7 ± 0.2
	DGN	45.5 ± 0.3	38.55 ± 0.3
	FUA	45.6 ± 0.05	37.95 ± 0.25

It is seen that CNTCOOHFUADGN@CSFISA had a superior anticancer effect, likely due to the presence of stearic acid, and may serve as DDS for this cell line. CNTNH_2_DGN also displayed a strong effect. DGN-loaded samples displayed a stronger effect than FUA-loaded ones.

The anticancer effect of the free natural agents and nanoformulations against A549 lung cancer cells was concentration-dependent (Figure 8B). The nanoformulations resulted in higher inhibition of A549 cell viability in comparison to free DGN and FUA (Table 5). These results demonstrate that CNTCOOHFUADGN@CSFISA and CNTNH_2_DGN greatly affected cell viability compared to other nanoformulations and free DGN and FUA.

In the case of MCF-7 breast cancer cells, the anticancer effect of nanoformulations was also significantly enhanced over that of free DGN and FUA (Figure 8C and Table 6). The effect was concentration-dependent. Greater inhibition of cell viability was observed when cells were treated with 2 μM. Table 6 indicates that FUA-loaded nanoformulations showed a stronger effect than the DGN-loaded ones. The anticancer effects were significantly stronger for single- or dual-loaded nanoformulations compared to free DGN and FUA.

**Table 6:** Shows the Ranking Effects on Cell Viability of MCF-7 Cells Depending on Nanoformulation Type

formulation	sample	viability % (MCF-7) cancer cells
F2	CNTCOOHFUA	9.0 ± 0.4
F5	CNTCOOHFUADGN	9.1 ± 0.3
F3	CNTNH_2_DGN	10.5 ± 0.4
F8	CNTCOOHFUADGN@CSFISA	12.0 ± 0.2
F4	CNTNH_2_FUA	13.1 ± 0.3
F1	CNTCOOHDGN	13.8 ± 0.3
F10	CNTNH_2_FUADGN@CSFISA	13.1 ± 0.3
F6	CNTNH_2_FUADGN	23.7 ± 0.4
	DGN	63.9 ± 0.2
	FUA	68.95 ± 0.2

Lower IC_50_ values were observed for noncoated nanoformulations compared to the coated ones, as well as for free DGN and FUA (Table S4 in the Supporting Information). For HepG2 cells, the following IC_50_ values were achieved: 0.34 mM/mL for CNTNH_2_FUADGN, 0.49 mM/mL for CNTCOOHFUADGN, 0.57 mM/mL for CNTCOOHFUADGN@CSFISA, and 0.61 mM/mL for CNTNH_2_FUADGN@CSFISA, respectively. The HepG2 and A549 cell lines exhibited higher sensitivity to the nanoformulations compared to the MCF7 cell line.

Three distinct effects were observed for the combined nanoformulations: synergistic, additive, and antagonist effects (Table S5, Supporting Information). The majority of the nanoformulations exhibited an additive effect, followed by synergistic and antagonist effects. It is worth noting that antagonist effects were primarily observed at higher concentrations, particularly at 2 mM/mL. CNTCOOHFUADGN displayed a synergistic effect when compared with other nanoformulations across all three cancer cell types. The natural substances FUA and DGN, when loaded on the CNTs, produce synergistic and additive effects for concentrations up to 1.5 mM/mL. For higher concentrations, antagonism effects are more prevalent.

To confirm the cellular morphology changes, we chose two nanoformulations: CNTCOOHFUADGN and CNTCOOHFUADGN@CSFISA, at 2 and 1.5 mM. We compared the morphology of cells in A549 cells before and after exposure to nanoformulations. Figure S4 and S5 shows the results. Untreated cancer cells had a consistent polygonal shape and were evenly dispersed throughout a cultivated area (Figure S6). Morphological changes were seen after 48 h of incubation with the nanoformulations, more expressed for CNTCOOHFUADGN than CNTCOOHFUADGN@CSFISA. The cells shrank after being exposed to the nanoformulations and changed their polygonal forms to circular ones. The untreated control cells exhibited no discernible morphological change.

**Figure 9:**
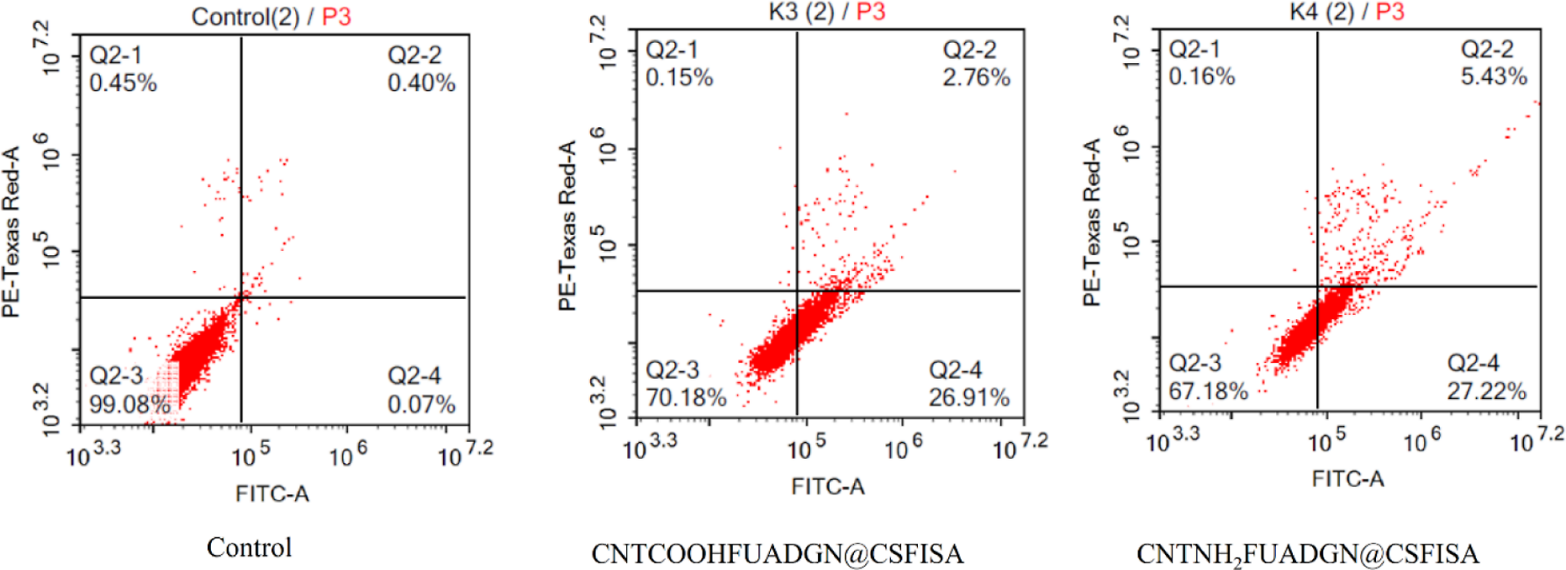
Evaluation of apoptosis induction in HepG2 cells following treatment with nanoformulations compared to control cells (without any treatments). Cells were treated with IC_50_ concentrations and incubated for 48 h. Cells were stained with PI/Annexin V-FITC. Q2-4 = early apoptosis, Q2-2 = late apoptosis, Q2-1 = necrosis, and Q2-3 = normal intact cells.

As depicted in Figure 9, to investigate apoptotic induction on HepG2 cells, the CNTCOOHFUADGN@CSFISA and CNTNH_2_FUADGN@CSFISA nanoformulations were tested. The total apoptosis (early and late) induced was 27.3 ± 1.2 and 2.6 ± 0.11 for CNTCOOHFUADGN@CSFISA and 26.8 ± 0.35 and 4.9 ± 0.43 for CNTNH_2_FUADGN@CSFISA. These findings make it evident that the apoptotic pathway causes cell death.

#### 3.3.3 Summary of Investigations of Biological Activity of the Formulations

The results obtained for the three cancer cell lines provide clear evidence that loading DGN or FUA into CNTs improves their anticancer activities compared to DGN or free forms. The amplitude of the effect depends on the concentration and cancer cell line. Similar results were observed for the targeted delivery system fabricated by loading the anticancer drug DOX into MWCNTs.^56^ Similar results have also been observed for PEGylated-MWCNTs incorporated with oxaliplatin, which remarkably improves the anticancer effect against HT-29 colon cells as a result of sustained release.^57^ In addition, functionalized MWCNT-NH_2_ loaded with the anticancer ruthenium(iii) drug exhibits higher anticancer efficiency against A549 cells, chronic myelogenous leukemia (K562) cells, and HeLa cells than the ruthenium complex itself.^58^ These results are consistent with nanoformulation delivery systems tailored by various nanostructures for DGN and FUA, improving the anticancer effects over the free forms of the drugs.^21−23,28−30^

Nanoformulations with stearic acid on the surface were more efficient against HepG2 and A549 cancer cells than against MCF-7 cells. This may be due to the positive impact of chitosan or stearic acid molecules on the endocytosis pathway through enhanced cellular uptake.^59^ The plausible explanation is that the cellular uptake considerably improved due to a chitosan–stearic acid coating interaction with cell membranes, allowing internalization and anticancer effects. Our findings demonstrate that the anticancer potential by cell line when ranked based on the lowest cell viability is as follows: HepG2 > A549 cells > MCF-7. Consequently, we further investigated HepG2 liver cancer cells to understand possible molecular targets.

### 3.4 Molecular Targets Evaluations

#### 3.4.1 Background for Molecular Targets Studies

lncRNAs are noncoding transcripts that revolutionized cancer treatment, offering a novel therapeutic approach for many tumors.^60^ They modulate pathways involving cell cycle, apoptosis, proliferation, invasion, and metastasis.^61−63^ lncRNAs play important roles in liver cancer progression, specifically hepatocellular carcinoma (HCC), showing both diagnostic and therapeutic potential.^64^ Many lncRNAs have been previously associated with liver cancer, including H19, HOTTIP, and HULC.^65,66^ All lncRNAs investigated in our study have been shown to be upregulated in liver cancers, allowing various functions.^64^ HULC enables tumor growth and a high rate of proliferation,^67^ HOTAIR promotes the proliferation of tumor cells,^68^ HOTTIP induces cell proliferation and viability,^69^ H19 is associated with cancer development, metastasis, and invasion,^70^ and CCAT promotes cancer progression.^71^

LncRNAs are involved in initiation, metastatic progression, and drug resistance via interactions with different miRNAs.^72^ Consequently, lncRNAs are recognition elements for miRNAs and affect their synthesis, maturation, and degradation.^73^ MiRNAs have a crucial role in the development of drug resistance in liver cancer.^74^ In our study, we investigated both tumor suppressors (mir-145 and mir-181a)^75^ and oncogenic miRNAs (mir-21 and mir-92).^76,77^ Mir-21 is overexpressed and identified as a key factor in tumor initiation, cell survival, the growth of liver cancer, and malignant development; inhibiting it leads to the induction of apoptosis.^78^ Tumor suppressor miRNAs are important for downregulating oncogene expression, as they can prevent the development and progression of liver cancer.^79^ Mir-145 is markedly decreased in liver cancer, and its restoration in HCC cells (HepG2 and Hep3B) permits the inhibition of proliferation and reduction of migration and invasion.^80^

The balance between oncogenes and tumor suppressors is key to sustaining the signals for cell survival and proliferation.^81^ TGF-β is a typical antiproliferative cytokine in epithelial cells, such as hepatocytes.^82^ TGF-β can switch to an oncogene to mediate cell survival and proliferation along with malignant progression in liver cancer.^83^ This issue was addressed in the present study. Although TGF-β has promising antiproliferative capability, it is also capable of tumor promotion; thus far, these dual actions are the major challenge in developing anticancer agents targeting the TGF-β pathway.^84^ We evaluated the expression of TGF-β and *E*-cadherin, both of which contribute to cancer progression and inhibitory effects.

*E*-Cadherin is a prototypical member of the type-1 classical cadherins and the major member of the cell adhesion molecule family expressed by epithelial cells.^85^*E*-Cadherin plays a role in the epithelial–mesenchymal transition (EMT), and its loss induces the EMT process that is associated with cancer stem cells and drug resistance in cancer.^86^ In addition, *E*-cadherin has regulatory effects on cell differentiation and maintains cell structure.^87^ For this reason, we evaluated *E*-cadherin expression in treated HepG2 cells compared with control cells.

To study the molecular targets of the constructed nanoformulations on the case of HepG2 cells, we selected the nanoformulations with dual agents (DGN and FUA) based on CNTCOOH of CNTCOOHFUADGN, CNTCOOHFUADGN@CSFISA (coated), and compared them with their free forms, as they showed the highest percentage of cell inhibition against this cell line. In Tables 5 and S4, all data are provided.

#### 3.4.2 Long Noncoding RNA Expression

**Figure 10:**
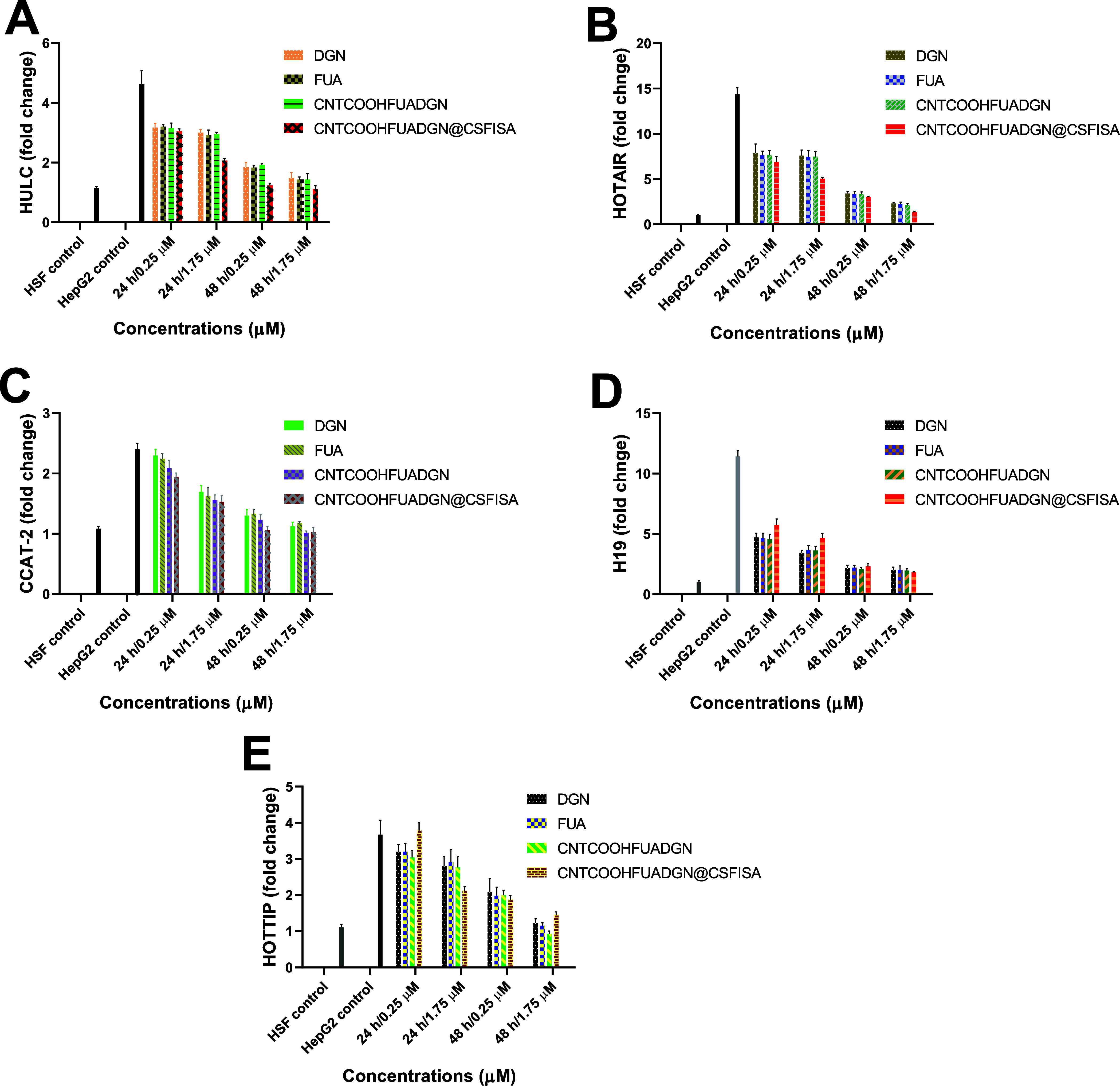
Long noncoding RNA expression assessed in normal HSFs, nontreated HepG2 cells (control), and HepG2 cells treated with nanoformulations and free DGN and FUA at different concentrations and incubation periods. (A) Expression of HULC lncRNA, (B) expression of HOTAIR lncRNA, (C) expression of CCAT-2 lncRNA, (D) expression of H19 lncRNA, and (E) expression of HOTTIP lncRNA. Data are provided as the mean ± SD, *N* = 3 replicates. H19 (*P* value: 0.0024, *P* value summary: **). Other markers were showed ns (non-significant).

Figure 10 shows the inhibition of all lncRNAs (HULC, HOTAIR, CCAT-2, H19, and HOTTIP) in HepG2 cells when treated with both nanoformulations and free DGN and FUA compared with both controls (HSFs and untreated HepG2 cells). The inhibition obtained was concentration-dependent and time-dependent. As expected, high inhibition was associated with an increased concentration and longer incubation time. Significant differences between nanoformulations and free agents were obtained only for the expression of H19 among all tested lncRNAs. The treatment of HepG2 cells at 1.75 μM CNTCOOHFUADGN@CSFISA for 48 h resulted in the highest HULC reduction (to 1.11 ± 0.10-fold change compared to 4.43 ± 0.40-fold change in untreated HepG2 control; Figure 10A). The inhibition of HULC with nanoformulations was relatively like that of normal cells (1-fold change). Similar results were obtained for HOTAIR expression (Figure 10B), and no significant differences were detected between DGN and FUA and nanoformulations. Inhibition was observed when HepG2 cells were exposed to CNTCOOHFUADGN@CSFISA at 1.75 μM for 48 h (1.35 ± 0.11-fold change) compared to that of untreated cells (14.36 ± 0.73-fold change). The change is 11 times lower when treating cells with nanoformulation than in untreated cells. As Figure 10C indicates, CCAT-2 expression was reduced by both nanoformulations at high concentration and longer incubation (48 h, ∼2.2-fold change) compared to control HepG2 cells (∼1.1-fold change). No significant differences were found between any of the treatments. Figure 10D shows that all treatments significantly decreased H19 expression in treated HepG2 cells compared to the control. CNTCOOHFUADGN@CSFISA resulted in better inhibition by increasing both concentration and incubation. It inhibited H19 (∼6-times) to 1.8 ± 0.1-fold change compared to 11.4 ± 0.46-fold change with control cells. The results for HOTTIP (Figure 10E) show that the maximum decrease observed when HepG2 cells were exposed to CNTCOOHFUADGN at 1.75 μM for 48 h resulted in a more than 3-fold change compared to that of control cells.

#### 3.4.3 MicroRNA Expression

**Figure 11:**
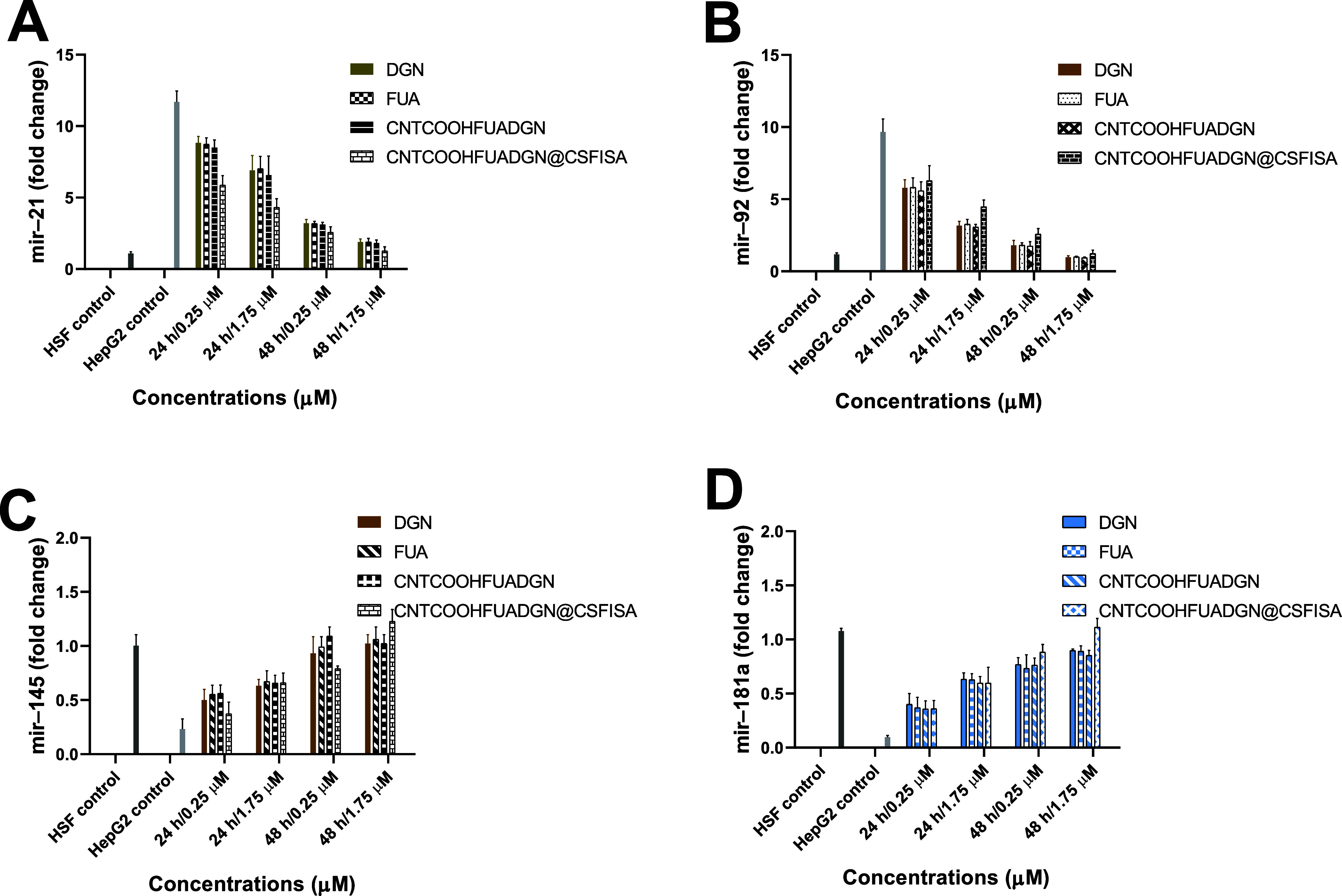
MicroRNA expression assessed in normal HSFs, nontreated HepG2 cells (control), and HepG2 cells treated with nanoformulations and free DGN and FUA at different concentrations and incubation periods. (A) Expression of mir-21, (B) expression of mir-92, (C) expression of mir-145, and (D) expression of 145a. Data are provided as the mean ± SD, *N* = 3 replicates. No significant differences detected between all samples for each tested miRNA at *P* < 0.05.

Figure 11 shows how the miRNA expression is affected by exposing HepG2 cells to both free DGN and FUA and nanoformulations. All miRNAs showed no significant differences among the tested samples. Both mir-21 and mir-92 were inhibited with increased concentration and longer incubation; in contrast, mir-145 and mir-181a gradually increased with increasing concentration and incubation compared with control HepG2 cells. Figure 11A shows that the maximum inhibition of mir-21 was detected for CNTCOOHFUADGN@CSFISA at <1.75 μM and 48 h, reaching more than a 9-fold change. Treating HepG2 cells with CNTCOOHFUADGN resulted in greater inhibition of mir-92 than other treatments, with approximately 8-times inhibition compared to the control (Figure 11B). In contrast, mir-145 and mir-181a gradually increased by increasing both concentration and time, and the greatest increase in mir-145 and mir-181a was detected by exposing cells to CNTCOOHFUADGN@CSFISA at 1.75 μM for 48 h. The increase in mir-145 reached a 1.22 ± 0.11-fold change compared to a 0.23 ± 0.09-fold change with the HepG2 control (Figure 11C), and mir-181a reached a 1.11 ± 0.08-fold change compared to a 0.09 ± 0.01-fold change with the HepG2 control (Figure 11D).

#### 3.4.4 Protein Expression

**Figure 12:**
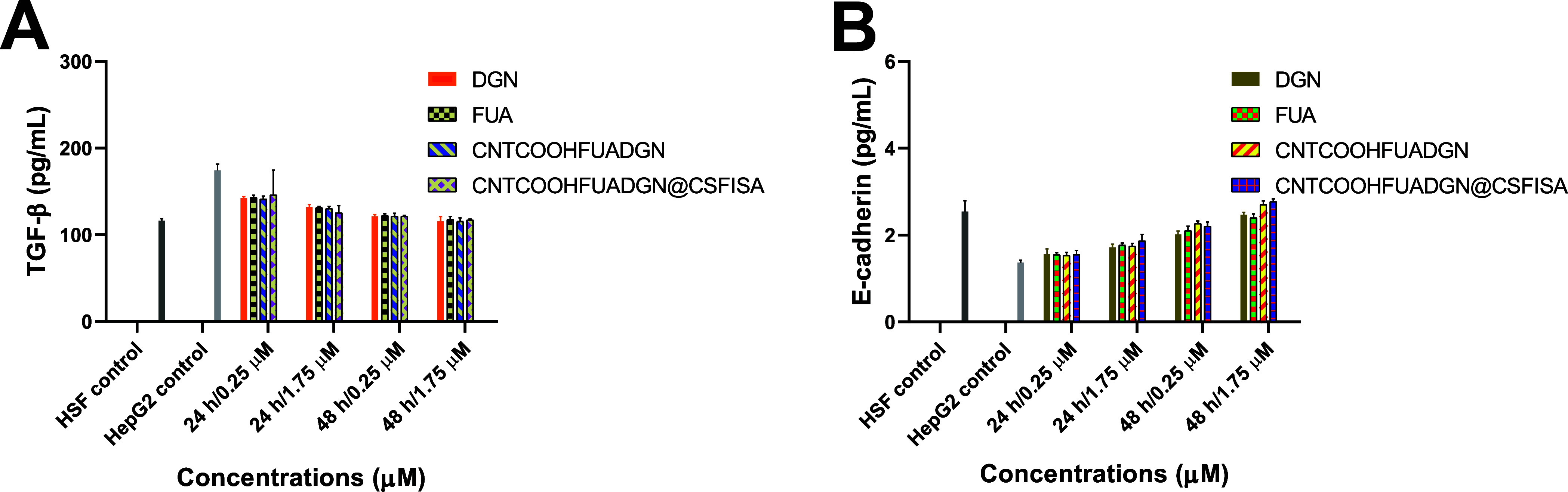
Protein expression assessed in normal HSFs, nontreated HepG2 cells (control), and HepG2 cells treated with nanoformulations and free DGN and FUA at different concentrations and incubation periods. (A) Expression of TGF-β protein and (B) expression of *E*-cadherin protein. Data are provided as the mean ± SD, *N* = 3 replicates. TGF-β (*P* value: 0.0357, *P* value summary: *). For *E*-cadherin, no significant differences were observed.

Figure 12A shows that the transforming growth factor beta (TGF-β) significantly (*p* < 0.05) decreased after treatment of HepG2 cells (∼117 pg/mL) compared to HepG2 control (174.33 ± 7.37 pg/mL). The maximum expression was obtained when cells were treated with CNTCOOHFUADGN and CNTCOOHFUADGN@CSFISA: the increase was approximately 2-times higher than the HepG2 control (Figure 12B).

### 3.5 Summary of Molecular Targets Evaluations

The investigated nanoformulations modulated the molecular targets of HepG2 cells significantly more strongly compared to free DGN and FUA, as indicated by the expression of lncRNAs, miRNAs, and proteins. The nanoformulations attenuate all five of the tested lncRNAs compared to those of untreated HepG2 liver cancer cells. The results are in agreement with our previous data demonstrating that free colchicine and nanoformulations both inhibit metastasis-associated lung adenocarcinoma transcript 1 (MALAT) lncRNA in HCT116 colon cancer cells.^39^

Improvement of mir-145 and mir-181a was found when cells were treated with CNTCOOHFUADGN and CNTCOOHFUADGN/CSFISA, compared to free pro-drugs. CNTCOOHFUADGN and CNTCOOHFUADGN/CSFISA could be effective in killing liver cancer cells by influencing miRNA expressions.^79^ Thus, they are promising for the prevention of drug-resistant liver cancer.

All treatments of free FUA and DGN and CNTCOOHFUADGN and CNTCOOHFUADGN/CSFISA nanoformulations decreased the level of TGF-β in HepG2 cells in a concentration-dependent and time-dependent manner. Therefore, it may be promising for promoting anticancer effects because TGF-β contributes to cancer progression and inhibitory effects.

Nanoformulations CNTCOOHFUADGN and CNTCOOHFUADGN/CSFISA induced higher *E*-cadherin expression in treated HepG2 cells than in control HepG2 cells and cells treated with free DGN and FUA. These findings could be of great importance and are in line with recent strategies to use pharmacologically small molecules to target the expression of *E*-cadherin in the treatment of liver cancer.^86^

## 4 Conclusions

DDSs for the delivery of FUA and DGN anticancer natural pro-drugs based on MWCNTs were produced. The procedure included functionalizing MWCNTs with COOH or NH_2_, subsequently loading them with FUA, DGN, or both, and finally coating them with chitosan or a mixture of chitosan and stearic acid. Particles in the form of entangled NTs with sizes ranging from 235 to 1100 nm, depending on the preparation method, and a negative surface charge were obtained. The MWCNTs could be loaded with the drugs in the range of 11–23% depending on the preparation method. The DGN and FUA release kinetics and total amount depend on GSH concentration, coating, and surface functionalization. The release occurred in two distinct stages: an initial zero-order release stage with no burst, followed by a progressively increasing sustained release pattern that best fits the Korsmeyer–Peppas kinetic model. The anticancer effect depends on cell line, with the order of effectiveness being HepG2 liver cells > A549 lung cancer > MCF7 breast cancer. The ranking of the nanoformulations concerning their anticancer effect is CNTNH_2_FUADGN > CNTCOOHFUADGN > CNTCOOHFUADGN@CSFISA > CNTNH_2_FUADGN@CSFISA according to their IC_50_. Combining the natural substances FUA and DGN into nanoformulations produces synergistic and additive effects. Experiments on HepG2 liver cancer cells indicated that the heightened anticancer effects could be attributed to the modulation of long noncoding RNAs (lncRNAs), microRNAs (miRNAs), and specific proteins. These findings provide valuable insights into the potential therapeutic benefits of natural agent-based nanoformulations in the context of cancer therapy.

## Data Availability

All data for this work is provided and available within this article, as well as supplementary material.

## Supporting Information Available

Size and zeta potential measurement of CNT materials and nanoformulations, detailed thermogravimetric characterization results, FTIR results, in vitro release data, IC_50_ data, calculated combination index, cellular morphology investigations on lung cells, methodology of flow cytometry studies, and molecular evaluations data (PDF)
