# Supplementary material for: Functionalized Carbon Nanotubes for Delivery of Ferulic Acid and Diosgenin Anticancer Natural Agents
Source: ACS Appl Bio Mater. 2024 Jan 22;7(2):791–811. doi: 10.1021/acsabm.3c00700 (PMC10880110; doi:10.1021/acsabm.3c00700)
Supplement: Supplementary file 1 — mt3c00700_si_001.pdf [file mt3c00700_si_001.pdf]

Supplementary material for the paper:

Functionalized carbon nanotubes for delivery of ferulic acid and diosgenin anticancer natural agents

Khaled AbouAitah[1]*, Ahmed M. Abdelaziz[2], Imane M. Higazy[3], Anna Swiderska-Sroda[4], Abeer M.E. Hassan[5], Olfat G. Shaker[6], Urszula Szałaj[4,7], Leszek Stobinski[8,9], Artur Malolepszy[9] and Witold Lojkowski[4]*

[1]Medicinal and Aromatic Plants Research Department, Pharmaceutical and Drug Industries Research Institute, National Research Centre (NRC), 33 El-Behouth Street, Dokki, Giza 12622, Egypt.

[2]Supplementary General Sciences, Future University, Cairo, Egypt.

[3]Department of Pharmaceutical Technology, Pharmaceutical and Drug Industries Research Institute, National Research Centre (NRC), 33 El-Behouth St, Dokki, Giza 12622, Egypt.

[4]Laboratory of Nanostructures and Nanomedicine, Institute of High Pressure Physics, Polish Academy of Sciences, Sokolowska 29/37, 01-142 Warsaw, Poland.

[5]Analytical chemistry department, Faculty of Pharmacy, October 6 University, Giza, Egypt.

[6]Medical Biochemistry and Molecular Biology Department, Faculty of Medicine, Cairo University, Cairo 11511, Egypt.

[7]Faculty of Materials Engineering, Warsaw University of Technology, Wołoska 41, 02-507 Warsaw, Poland.

[8] NANOMATPL Ltd., 14/38 Wyszogrodzka Str., Warsaw, Poland

[9]Faculty of Chemical and Process Engineering, Warsaw University of Technology, 1 Warynskiego St., 00-645, Warsaw, Poland

* Correspondence:

Correspondence: e-mail: ke.abouaitah@nrc.sci.eg; Tel.: +20233371635; Fax: +20233371010 (K.A.); w.lojkowski@labnano.pl (W.L.); Tel.: +48-22-888-0429 or +48-22-632-4302; Fax: +48-22-632-4218 (W.L.)

1. **Study of size distribution of nanoparticles and zeta potential**

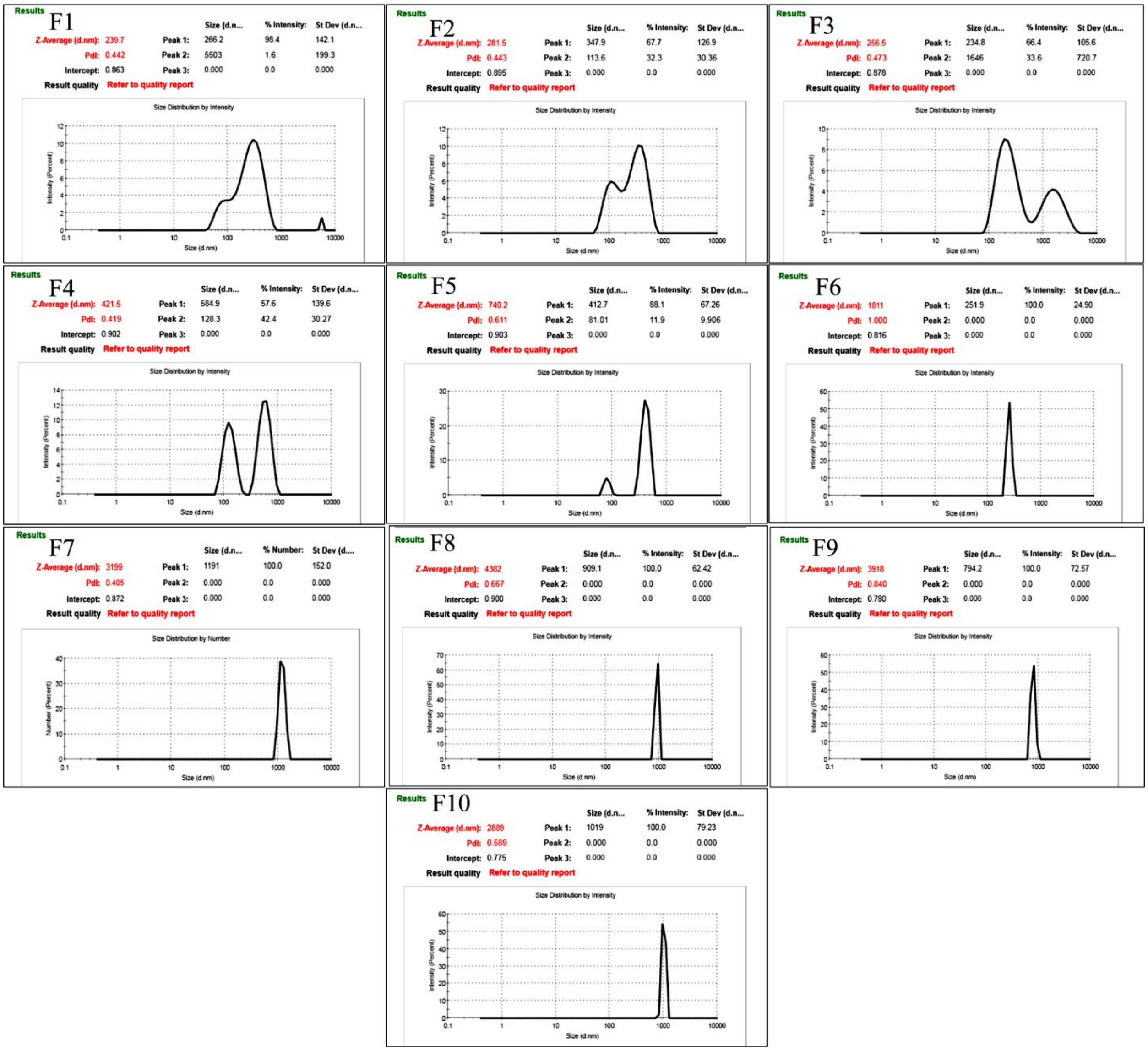

Figure S1. Size distribution measurement of the samples as evaluated using the DLS method. The samples description is given in Table 1

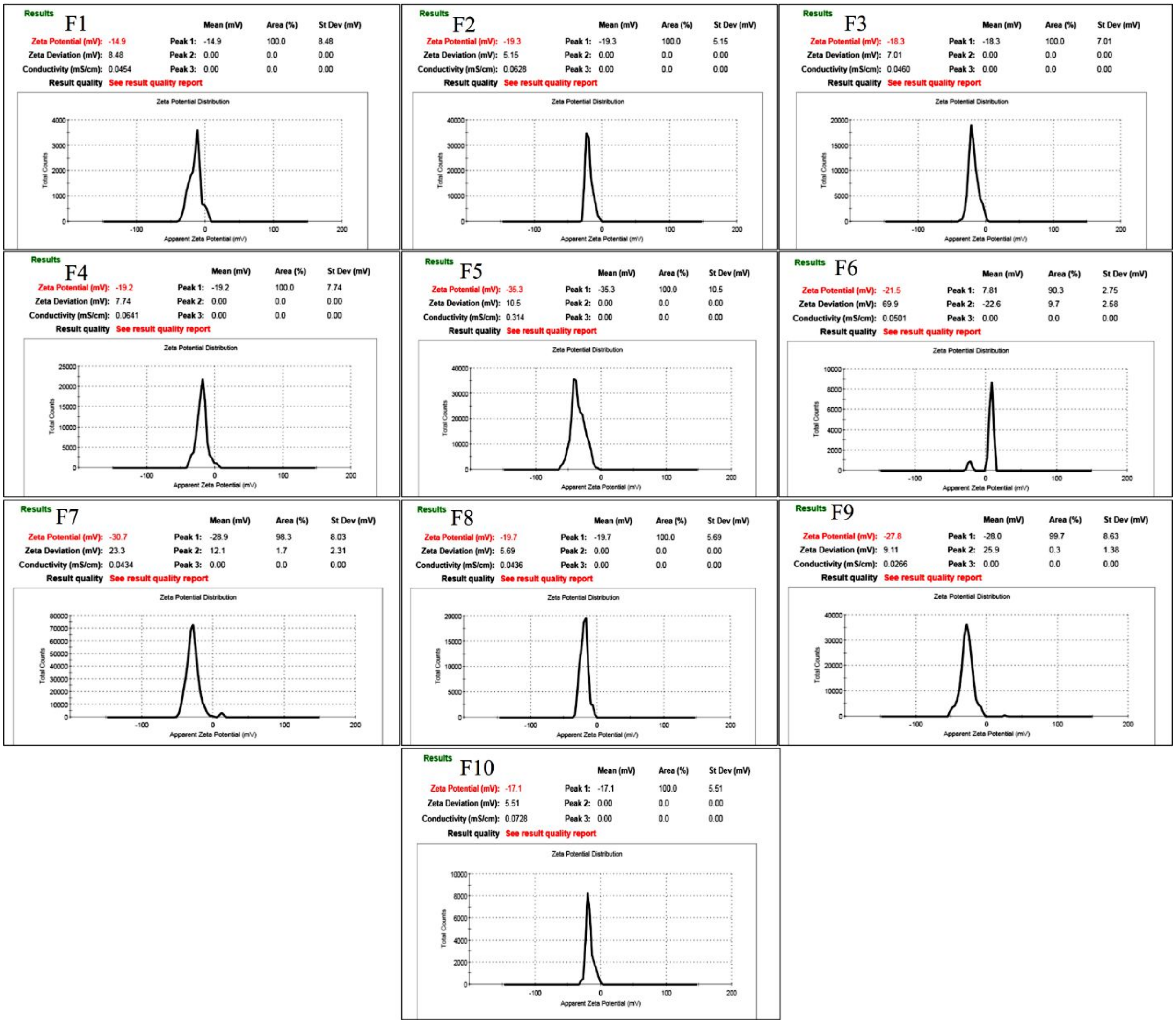

Figure S2. Zeta potential measurement of CNT materials and nanoformulations. The meaning of the symbols F1-F10 are explained in Table S1.

Table S1: Mean size and zeta potential of the samples.

Sample	Size of the lager peak, nm	Size of the smaller peak, nm and % of intensity	Zeta potential , mV
F1	266 ± 142	-	-14.9 ± 8.5
F2	348 ± 127	113 ± 32 (32%)	-19,3 ± 5,1
F3	235 ± 105	1664 ± 720 (33%)	-18,3 ± 7.0
F4	585 ± 140	128 ± 30 (42,4)%	-19.2 ± 7.7
F5	413 ± 68	81 ± 10 (12%)	-35.3 ± 10.5

F6	250 ± 25	-	-21,5 ± 2.7
F7	1191 ± 152	-	-28.7 ± 8.3
F8	909 ± 62	-	-19.7 ± 5.7
F9	792 ± 73	-	-28.0 ± 8.6
F10	1109 ± 79	-	-17.1 ± 5.5

It is seen that the non-coated with polymer samples show a bi-modal size distribution, except for the F6 - $CNTNH_2FUA$ sample. The coated samples show significantly larger size than the non-coated samples.

For interpretation of these results is to consider that the DLS method is not suitable for samples of fibrous structure, as CNTs. The size of such samples depends rather on their length than diameter and in addition, the fibres are entangled, as seen on FE-SEM images (Fig 1 in the main text). However, large size of the coated samples may indicate their agglomeration. The samples without polymer coating display a mean size in the range 235 – 585 nm, while the coated ones in the range 792 – 1191 nm.

No systematic trend is seen as far as the Zeta-potential is concerned.

2. Thermogravimetric characterization

Pure DGN and FUA decomposed nearly completely (mass losses of 98 wt.% and 92 wt. %, respectively). Supplementary material describes in detail decomposition of these substances. Intensive weight losses related to decomposition are visible on the TG curves in the temperature range of 250-560°CC for DGN and 170-430℃ for FEA (**Figure S3A,B**). The corresponding broad peaks in the DTG curves have extremes at approximately 350°C and 250°C, respectively (**Figure S3C,D**). The DSC curves of both anticancer agents were similar in nature (**Figure S3E,F**). Only endothermic peaks are visible. The first peak was intense and narrow. For DGN, it occurred between 180 and 220℃, with a minimum of approximately 205℃. For FUA, the peak occurred in the range of 160-180℃ with a minimum at approximately 175℃. This thermal effect was not accompanied by a change in the mass of the substance (no signals on the DTG curve). These peaks are related to the melting process of DGN and FUA [1, 2]. At higher temperatures, wide endothermic signals were present in DSC curves of DGN and FUA, correlating with the decomposition process. In the case of DGN, a series of overlapping peaks occur in the temperature

range of 290-560°C. The most intense peak had a minimum at approximately 360°C. For FUA, the endothermic effect was visible at 200-280°C with a minimum at approximately 260°C.

We did not find any significant differences between the non-coated nor loaded two types of nanotubes of CNTCOOH and $CNTNH_2$. The weight losses of CNTOOH and $CNTNH_2$ reached 15.6 wt. % and 17.6 wt. %, respectively (**Figure S3A, B**). Thus, the amount of functionalizing substance/number of groups on the CNT surface is insignificant, at approximately 2 wt.%. The TG curves of CNTOOH and $CNTNH_2$ followed a similar course. At the temperature range of RT-160°C, we observed a slight change in the kinetics of the mass loss, which corresponds to a broad, weak peak on the DTG curves (**Figure S3C,D**). This is most likely due to surface dehydration of the CNTs. A further temperature increase causes a uniform, systematic mass decrease in CNTCOOH. In the case of CNT-NH_2, an additional slight inflection in the TG curve was observed and an adequate wide, weak peak on the DTG curve in the range of 400-560°C. This is probably the signal related to decomposition of the amino groups situated on the CNT surface. No thermal effects are observed in the DSC curves for both types of nanotubes (**Figure S3E,F**). The lack of a signal related to the decomposition of the APTES groups in CNT-NH_2 is most likely due to the small amount of modifier (approx. 2 wt.%). Thermal analysis of nanoformulations confirmed that the steps following CNT modification were a success. The weight loss of DGN and FUA-enriched nanotubes doubled in relation to unmodified CNTs. After additional chitosan-stearic acid coating, the weight loss was 3-times greater. Mass losses by nanoformulations based on CNTCOOH reached approximately 32 wt.% for CNTCOOHDGN, 38 wt.% for CNTCOOHFUA, 38 wt.% for CNTCOOHFUADGN, and 57 wt.% for CNTCOOHFUADGN@CSFISA. In the case of nanoformulations prepared with $CNTNH_2$, the mass losses reached approximately 34 wt.% for $CNTNH_2DGN$, 18 wt.% for $CNTNH_2FUA$ (similar to $CNTNH_2$ before loading, which may be related to interaction of FUA and CNTs), 36 wt.% for $CNTNH_2FUADGN$, and 57 wt.% for $CNTNH_2FUADGN$@CSFISA.

The significant differences in thermal properties between non-loaded and loaded materials is related to decomposition of the anticancer agents and chitosan-stearic acid coating complex. The temperature range of the decomposition process was determined from the peaks visible in the DTG curves (**Figure S3C,D**). In the case of nanotubes with DGN, the temperature corresponded to the decomposition of the pure anticancer substance: 160-520°C (min. at 290°C) for CNTOOHDGN and 200-560°C (min. at 300°C) for $CNTNH_2DGN$. In the case of CNTs with FUA, the results were not so evident. Intensive mass loss of CNTOOHFUA occurred at lower temperatures than for pure

FUA. The DTG peak was presented at RT-220°C with a minimum at 110°C. The CNT-NH_2FUA results were very similar to those obtained for pure CNT-NH_2. The DTG curves for nanotubes enriched with two active agents exhibited the decomposition effects of both DGN and FUA. For CNTOOHFUADGN, the first occurred at RT-160°C (min. ~120°C) and second at 180-480°C (min. ~290°C). For CN-NH2FUADGN, the first occurred at 210-350°C (min. ~300°C) and second at 350-500°C (min. at 420°C). The TG/DTG curves of both nanoformulations with the polymer coating had a similar course (**Figure S3A-D**). The broad peaks in the DTG curves, corresponding to the FUA and DGN decomposition, occurred in the range of RT-160°C (min. ~95°C) and 160-400°C (min. ~250°C), respectively. For CNTCOOHFUADGN@CSFISA, an additional peak was visible in the range 350-520°C (min. at 430°C). For all investigated nanoformulations, there were no melting peaks, which were recorded for pure anti-cancer substances (**Figure S3E,F**). The reason may be the π–π interaction of nanotubes and drugs [3], an insufficient amount of natural agent, and changes in the structure of drugs during the preparation of the nanoformulations.

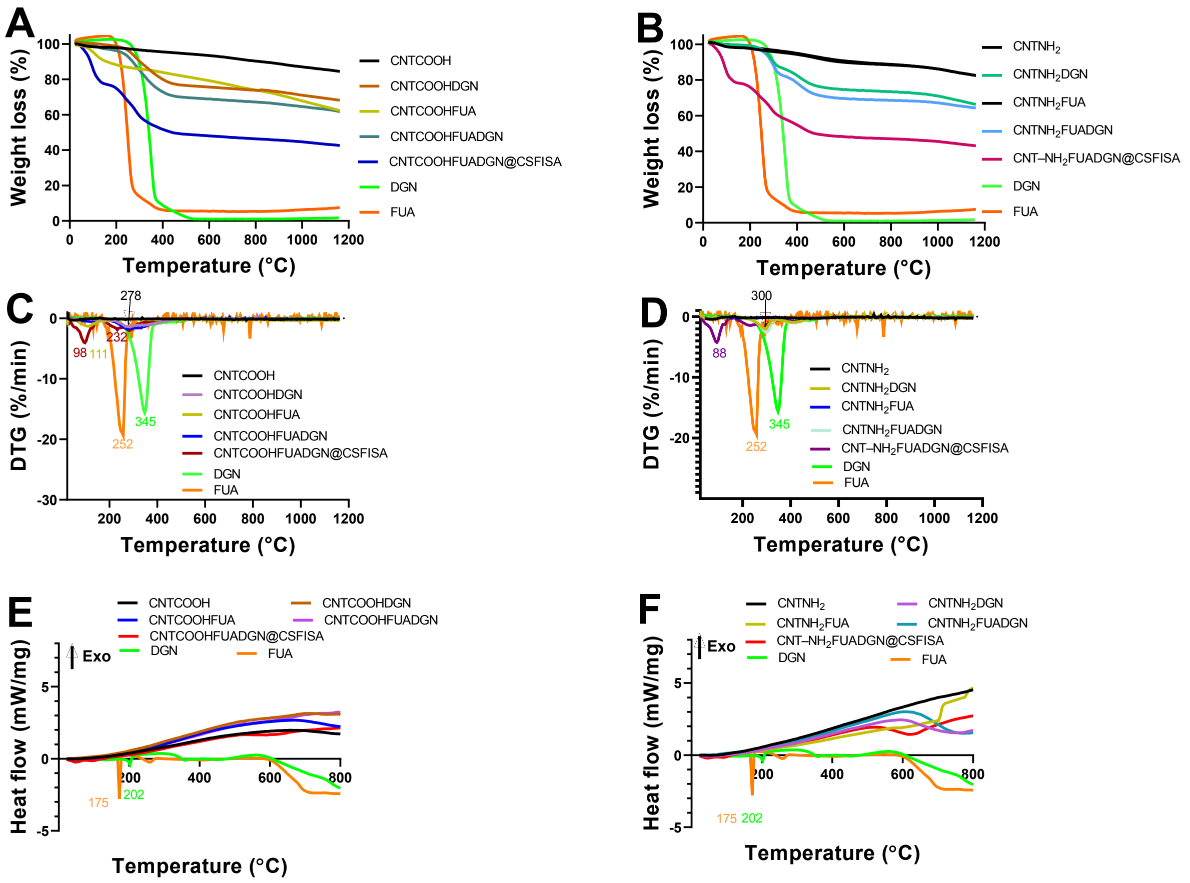

Figure S3. Thermal characterization of CNTs, nanoformulations, and free natural agents. (A, B) Weight loss measurements by simultaneous thermal analysis. (C, D) DTG curves. (E, F) Differential scanning calorimetry thermograms.

3. FTIR results

Bands corresponding to hydroxyl groups at approximately 3400 cm^{-1} and a number of signals at lower wavenumbers [1, 4]. In the case of DGN, the band assigned to the -OH stretching vibrations occurred at 3447 cm^{-1} and the peaks attributed to the CH_2 stretching and scissoring vibrations at 2950 cm^{-1} and 1455 cm^{-1}, respectively. Bands corresponding to -C-O stretching vibrations were visible at 1172 cm^{-1} and 1051 cm^{-1}, whereas those corresponding to the CH_2 twisting vibrations were visible at 896 cm^{-1} [5]. FUA showed a signal corresponding to hydroxyl groups (-OH) at 3432 cm^{-1}, the alkane characteristic band at 1687 cm^{-1}, C=C stretching vibration at 1661 cm^{-1}, -C-O stretching vibrations at 1163 cm^{-1}, and C=C-H bond at 942 cm^{-1} [4].

The results obtained for both types of nanotubes were very similar, with no intense well-defined signals detected.

4. In vitro release data.

Table S2. In vitro release data.

Time (h)	No GSH		GSH (10%)		GSH (20%)	
	F1	F2	F1	F2	F1	F2
1	**0.15 ± 0.01**	**0.18 ± 0.00**	**0.29 ± 0.01**	**0.23 ± 0.01**	**0.46 ± 0.03**	**0.37 ± 0.01**
2	**0.68 ± 0.03**	**0.51 ± 0.01**	**0.94 ± 0.02**	**0.70 ± 0.02**	**1.25 ± 0.13**	**0.86 ± 0.02**
3	**1.32 ± 0.03**	**0.99 ± 0.03**	**2.05 ± 0.12**	**1.30 ± 0.08**	**3.38 ± 0.80**	**2.26 ± 0.17**
4	**3.69 ± 0.12**	**2.87 ± 0.10**	**6.13 ± 0.84**	**3.19 ± 0.16**	**7.28 ± 2.09**	**4.30 ± 0.28**
6	**7.50 ± 0.50**	**6.85 ± 0.48**	**11.42 ± 0.99**	**8.54 ± 2.01**	**13.70 ± 2.55**	**10.52 ± 1.00**
8	**12.11 ± 1.35**	**13.03 ± 0.95**	**18.16 ± 0.95**	**17.68 ± 1.18**	**20.85 ± 2.98**	**19.75 ± 1.21**
12	**23.44 ± 1.76**	**24.32 ± 0.99**	**27.26 ± 2.12**	**28.97 ± 1.80**	**32.15 ± 1.79**	**30.14 ± 1.99**
24	**38.73 ± 1.98**	**33.75 ± 2.13**	**42.01 ± 3.06**	**38.08 ± 2.74**	**46.31 ± 1.25**	**40.60 ± 3.32**
36	**62.68 ± 3.37**	**50.43 ± 1.45**	**70.10 ± 5.21**	**56.99 ± 4.06**	**75.16 ± 3.16**	**59.93 ± 3.98**
48	**78.23 ± 5.80**	**71.20 ± 3.62**	**83.19 ± 3.80**	**77.15 ± 4.25**	**88.17 ± 3.71**	**80.07 ± 4.22**
72	**90.31 ± 3.99**	**88.21 ± 3.18**	**94.60 ± 5.10**	**95.11 ± 2.91**	**96.41 ± 2.66**	**98.12 ± 1.93**
R2	**0.9892**	**0.9888**	**0.9927**	**0.9902**	**0.9951**	**0.9935**
KM	**Korsmeyer-Peppas**		**Korsmeyer-Peppas**		**Korsmeyer-Peppas**	

F1 (CNT-NH_2-DGN)
F2 (CNT-COOH-DGN)

Time (h)	No GSH		GSH (10%)		GSH (20%)	
	F3	F4	F3	F4	F3	F4
1	0.21 ± 0.01	0.13 ± 0.01	0.30 ± 0.01	0.26 ± 0.01	0.44 ± 0.02	0.34 ± 0.01
2	0.56 ± 0.01	0.42 ± 0.03	0.63 ± 0.03	0.51 ± 0.01	0.91 ± 0.01	0.60 ± 0.02
3	1.39 ± 0.05	1.18 ± 0.01	1.88 ± 0.06	2.01 ± 0.01	2.53 ± 0.18	2.45 ± 0.02
4	4.55 ± 0.18	3.97 ± 0.11	6.01 ± 0.59	5.15 ± 0.57	8.91 ± 0.33	6.70 ± 0.42
6	12.19 ± 1.00	10.21 ± 0.98	14.62 ± 1.25	13.18 ± 0.90	19.47 ± 1.29	17.64 ± 0.89
8	20.07 ± 1.12	17.89 ± 1.05	25.14 ± 1.03	20.07 ± 1.34	30.01 ± 2.08	27.82 ± 1.47
12	32.20 ± 1.92	28.68 ± 1.88	38.12 ± 2.11	30.20 ± 2.21	42.38 ± 1.72	36.99 ± 1.59
24	45.18 ± 3.16	40.16 ± 3.30	54.77 ± 2.82	45.61 ± 2.63	59.17 ± 3.45	51.08 ± 2.20
36	66.34 ± 2.17	62.55 ± 2.19	70.92 ± 4.19	69.72 ± 4.01	76.86 ± 2.19	73.13 ± 1.99
48	79.18 ± 2.99	72.90 ± 3.70	84.16 ± 3.15	77.85 ± 3.60	90.21 ± 4.31	83.19 ± 2.16
72	92.73 ± 1.28	89.72 ± 3.82	95.03 ± 2.62	92.44 ± 2.22	98.23 ± 2.10	96.11 ± 1.86
**R2**	0.9878	0.9845	0.9890	0.9892	0.9912	0.9894
**KM**	Korsmeyer-Peppas		Korsmeyer-Peppas		Korsmeyer-Peppas	

F3 (CNT-NH_2-FeA)

F4 (CNT-COOH-FeA)

Time (h)	No GSH		GSH (10%)		GSH (20%)	
	F5A (DGN)	F5B (FeA)	F5A (DGN)	F5B (FeA)	F5A (DGN)	F5B (FeA)
1	0.11 ± 0.00	0.16 ± 0.01	0.20 ± 0.02	0.24 ± 0.01	0.36 ± 0.03	0.41 ± 0.01
2	0.47 ± 0.02	0.51 ± 0.02	0.58 ± 0.04	0.93 ± 0.05	0.78 ± 0.05	1.20 ± 0.02
3	0.82 ± 0.02	1.12 ± 0.07	1.27 ± 0.08	3.00 ± 0.18	2.34 ± 0.14	4.14 ± 0.25
4	1.60 ± 0.10	3.83 ± 0.16	3.46 ± 0.19	4.55 ± 0.31	5.60 ± 0.28	6.70 ± 0.53
6	5.15 ± 0.24	9.11 ± 0.44	7.72 ± 0.35	11.79 ± 0.93	9.13 ± 0.58	16.04 ± 0.85
8	9.84 ± 0.56	15.58 ± 0.96	12.05 ± 0.91	19.26 ± 1.40	16.37 ± 1.02	28.92 ± 1.66
12	15.39 ± 1.02	26.21 ± 1.54	20.18 ± 0.85	30.53 ± 2.29	38.50 ± 2.10	36.02 ± 1.59
24	29.41 ± 1.53	40.81 ± 1.99	34.01 ± 1.07	43.90 ± 3.11	42.24 ± 1.80	49.16 ± 3.20
36	45.20 ± 3.12	58.07 ± 2.68	50.33 ± 3.50	65.51 ± 2.75	60.94 ± 2.40	70.31 ± 3.16
48	63.77 ± 2.98	74.43 ± 2.53	71.19 ± 2.49	79.39 ± 2.15	78.39 ± 4.66	85.46 ± 2.23
72	86.95 ± 3.74	89.30 ± 4.71	90.55 ± 2.30	93.99 ± 2.78	94.02 ± 1.92	97.15 ± 1.39
R2	0.9955	0.9872	0.9909	0.9920	0.9930	0.9936
KM	Zero Order	Korsmeyer-Peppas	Korsmeyer-Peppas		Korsmeyer-Peppas	

F5A CNT-NH2-FeA-DGN (as DGN)

F5B CNT-NH2-FeA-DGN (as FeA)

Time (h)	No GSH		GSH (10%)		GSH (20%)	
	F6A (DGN)	F6B (FeA)	F6A (DGN)	F6B (FeA)	F6A (DGN)	F6B (FeA)
1	0.06 ± 0.00	0.10 ± 0.00	0.15 ± 0.01	0.20 ± 0.01	0.25 ± 0.01	0.34 ± 0.01
2	0.14 ± 0.01	0.29 ± 0.00	0.32 ± 0.00	0.73 ± 0.04	0.55 ± 0.01	1.04 ± 0.09
3	0.23 ± 0.01	1.00 ± 0.07	0.99 ± 0.04	2.61 ± 0.09	1.75 ± 0.09	3.85 ± 0.15
4	1.01 ± 0.03	2.81 ± 0.05	2.50 ± 0.12	3.89 ± 0.16	4.30 ± 0.20	5.39 ± 0.37
6	3.78 ± 0.10	7.76 ± 0.30	5.24 ± 0.25	9.40 ± 0.59	7.90 ± 0.51	14.20 ± 0.91
8	6.11 ± 0.18	13.16 ± 0.97	9.80 ± 0.34	17.07 ± 1.03	14.08 ± 1.04	22.62 ± 1.13
12	10.45 ± 0.96	21.10 ± 1.56	14.91 ± 1.11	27.11 ± 1.41	29.44 ± 1.31	33.94 ± 2.10
24	19.61 ± 1.03	32.09 ± 2.02	26.05 ± 1.87	38.05 ± 1.50	38.21 ± 1.94	45.72 ± 1.20
36	33.52 ± 1.77	44.88 ± 1.99	41.11 ± 2.51	60.51 ± 3.64	54.13 ± 3.73	68.02 ± 4.38
48	55.37 ± 3.01	64.93 ± 4.30	62.58 ± 1.99	72.35 ± 2.13	70.02 ± 2.66	80.81 ± 1.94
72	77.82 ± 2.80	83.54 ± 3.01	80.20 ± 3.09	90.12 ± 2.29	91.49 ± 1.17	95.31 ± 2.05
R2	0.9907	0.9849	0.9907	0.9911	0.9911	0.9933
KM	Zero Order	Zero Order	Zero Order	Korsmeyer-Peppas	Korsmeyer-Peppas	

F6A CNT-COOH-FeA-DGN (as DGN)

F6B CNT-COOH-FeA-DGN (as FeA)

Time (h)	No GSH		GSH (10%)		GSH (20%)	
	F7A (DGN)	F7B (FeA)	F7A (DGN)*	F7B (FeA)	F7A (DGN)	F7B (FeA)
1	0.23 ± 0.00	0.33 ± 0.01	0.30 ± 0.02	0.41 ± 0.01	0.43± 0.02	0.65 ± 0.04
2	0.46 ± 0.03	0.54 ± 0.01	0.53 ± 0.02	0.66 ± 0.03	0.79 ± 0.05	0.94 ± 0.02
3	0.89 ± 0.03	0.92 ± 0.05	1.14 ± 0.07	1.22 ± 0.08	1.88 ± 0.10	2.03 ± 0.15
4	1.78 ± 0.10	2.15 ± 0.18	3.76 ± 0.16	4.61 ± 0.22	6.13 ± 0.28	6.58 ± 0.42
6	5.21 ± 0.37	9.32 ± 1.00	7.99 ± 0.62	14.27 ± 1.01	10.61 ± 0.95	17.72 ± 1.06
8	10.92 ± 0.86	18.35 ± 1.12	15.61 ± 1.01	25.82 ± 2.21	22.39 ± 1.14	32.22 ± 2.39
12	21.94 ± 2.03	28.82 ± 1.92	32.24 ± 1.44	37.84 ± 1.73	40.53 ± 1.32	47.10 ± 1.75
24	35.80 ± 2.66	43.12 ± 3.16	43.58 ± 1.87	51.30 ± 3.66	54.18 ± 2.16	60.82 ± 2.90
36	53.11 ± 1.80	57.49 ± 2.17	58.30 ± 1.55	65.63 ± 1.80	65.11 ± 3.30	71.53 ± 4.26
48	64.60 ± 4.93	71.63 ± 2.99	70.08 ± 1.96	78.20 ± 4.15	76.23 ± 2.51	82.30 ± 2.71
72	72.22 ± 2.44	80.20 ± 1.28	80.42 ± 3.49	85.55 ± 2.97	87.47 ± 3.29	90.41 ± 2.35
R2	0.9894	0.9888	0.9899	0.9894	0.9924	0.9937
KM	Korsmeyer-Peppas		Korsmeyer-Peppas		Baker-Lonsdale	

F7A CNT-NH2-FeA-DGN@CS/SA (as DGN)

F7B CNT-NH2-FeA-DGN@CS/SA (as FeA)

Time (h)	No GSH		GSH (10%)		GSH (20%)	
	F8A (DGN)	F8B (FeA)	F8A (DGN)	F8B (FeA)	F8A (DGN)	F8B (FeA)
1	0.09 ± 0.00	0.20 ± 0.01	0.16 ± 0.01	0.31 ± 0.00	0.27 ± 0.01	0.55 ± 0.02
2	0.17 ± 0.00	0.33 ± 0.01	0.25 ± 0.01	0.45 ± 0.02	0.43 ± 0.03	0.70 ± 0.02
3	0.34 ± 0.01	0.79 ± 0.05	0.67 ± 0.03	0.98 ± 0.04	0.98 ± 0.01	1.83 ± 0.07
4	0.59 ± 0.03	1.95 ± 0.11	1.81 ± 0.14	3.62 ± 0.11	2.43 ± 0.09	5.13 ± 0.25
6	3.20 ± 0.14	5.72 ± 0.25	4.70 ± 0.25	10.01 ± 0.92	8.62 ± 0.52	14.45 ± 0.94
8	6.13 ± 0.43	10.90 ± 0.72	8.08 ± 0.62	17.59 ± 1.02	17.28 ± 0.76	28.51 ± 1.12
12	11.52 ± 1.07	21.32 ± 1.31	18.10 ± 1.06	30.36 ± 1.31	30.99 ± 2.05	40.77 ± 1.58
24	27.12 ± 1.23	35.11 ± 2.29	30.01 ± 1.55	46.27 ± 2.15	45.57 ± 1.93	57.06 ± 1.66
36	40.68 ± 3.60	49.52 ± 3.51	44.24 ± 1.72	60.15 ± 3.06	59.16 ± 2.41	68.93 ± 2.03
48	58.85 ± 2.22	62.31 ± 2.13	64.71 ± 2.50	74.88 ± 3.29	72.04 ± 2.12	79.99 ± 2.99
72	65.30 ± 1.85	70.28 ± 1.52	75.99 ± 1.93	80.12 ± 1.02	81.17 ± 1.11	87.20 ± 1.34
R2	0.9782	0.9874	0.9858	0.9882	0.9874	0.9911
KM	Korsmeyer-Peppas		Korsmeyer-Peppas		Korsmeyer-Peppas	

F8A CNT-COOH-FeA-DGN@CS/SA (as DGN)

F8B CNT-COOH-FeA-DGN@CS/SA (as FeA)

4. Biological evaluations

Table S4. IC50 values for single and combined nanoformulations, as well as free DGN and FUA.

	**IC50 (mM/mL)/Nanoformulations**									
**Cell type**	F1	F2	F3	F4	F5	F6	F7	F8	DGN	FUA
HepG2 cells	1.37	0.71	1.48	0.57	1.05	0.85	0.34	0.61	0.74	1.15
A545 cells	1.27	0.925	0.491	1.174	0.793	1.05	0.68	0.82	1.42	0.83
MCF7 cells	1.26	1.21	0.98	1.14	0.99	1.47	0.98	1.27	1.02	1.3

F1= CNTCOOHDGN, F2= CNTCOOHFUA, F3= CNTCOOHFUADGN, F4= CNTCOOHFUADGN@CSFISA, F5= CNTNH2DGN, F6= CNTNH2FUA, F7= CNTNH2FUADGN, F8= CNTNH2FUADGN@CSFISA.

EC50(IC50) was computed with values of cell inhibition% by origin in dose response fitting.

Table S5. Calculated combination index for single nanoformulations composed FUA or DGN and combined nanoformulation composed both agents before and after polymeric shell coating.

**Cell type**	**Conc.**	**Combination index by high single agent model (HAS)**			
	(uM/mL)	CNTCOOHFUADGN	CNTCOOHFUADGN@CSFISA	CNTNH2FUADGN	CNTNH2FUADGN@CSFISA
HepG2	0.25	31.54243	22.65758	3.35769	-48.84773
	0.75	23.95231	15.773	2.5668	-62.88974
	1.5	10.36923	1.31923	-15.8023	-17.41038
	2	-2.521952	1.301396	-2.959197	-9.807797
A549	0.25	14.97625	10.04355	5.01923	-39.47439
	0.75	23.86154	-4.13846	-7.74539	-26.49154
	1.5	13.83461	8.93461	-4.28847	-42.11154
	2	1.23077	2.2	-6.03077	-8.66154
MCF7	0.25	12.07307	1.85	4.16538	-48.99231
	0.75	15.90385	5.34615	6.55769	-40.02693
	1.5	10.9923	3.11538	-2.05	-24.98846
	2	-0.119231	-2.988464	-13.14615	-3.70384

Synergy: > 10
Antagonism effect: less than - 10
Additive effect: from -10 to 10

To calculate CI, we do the following steps:

1. The calculation was performed with cell inhibition percent%.

As our data presented in the paper in cell viability %, we firstly calculated the cell inhibition according to this equation:

Cell inhibition %= 100 – cell viability.

2. For CI calculation, we used HAS combination index calculation eq:

CI= EAB–max(EA,EB)

EAB= effect producing cell inhibition% of combined two drugs (FUA combined DGN) into nanoformulation before and after polymeric coating.

EA and EB = effect producing cell inhibition% of single drug (FUA or DGN) into nanoformulation.

5. Morphology investigations

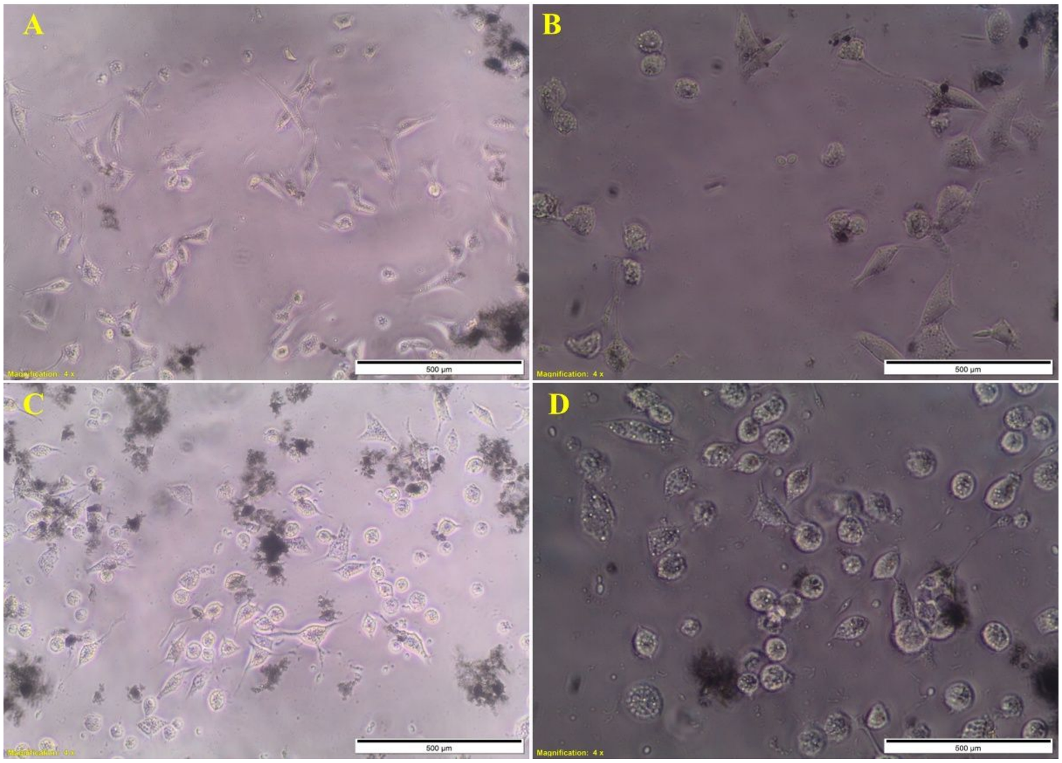

Figure S4. Morphological observation of A549 lung cancer cells after their treatment with CNTCOOHFUADGN nanoformulation. A&B) cells treated with 2 mM. C&D) cells treated with 1.5 mM.

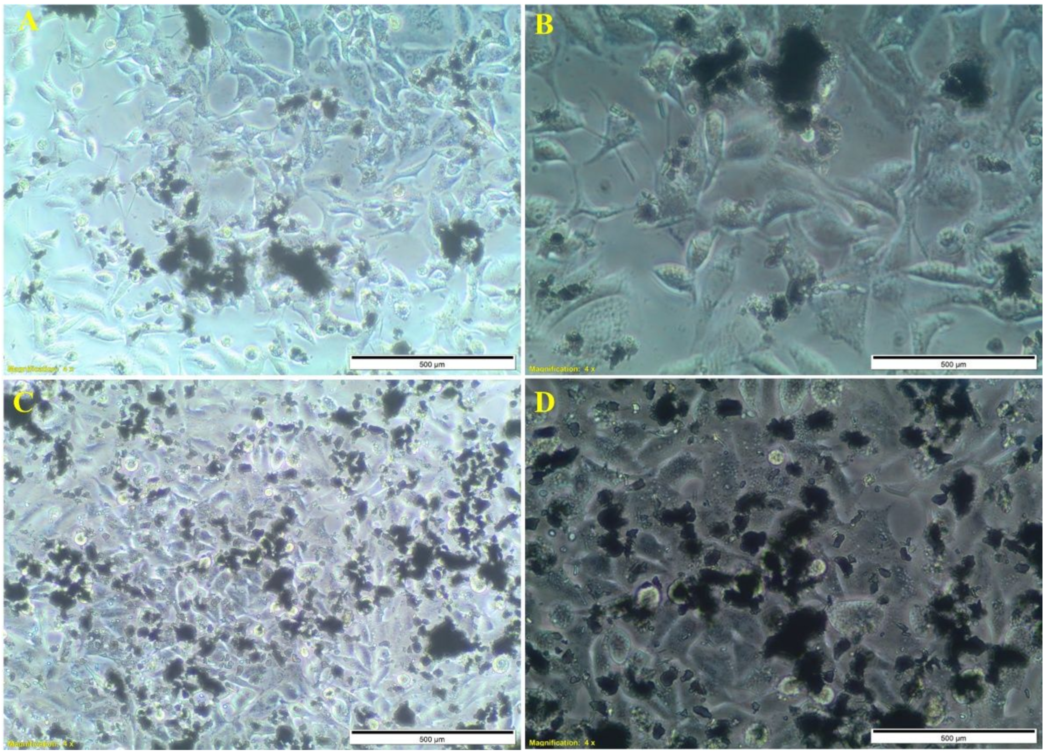

Figure S5. Morphological observation of A549 lung cancer cells after their treatment with CNTCOOHFUADGN@CSFISA nanoformulation. A&B) cells treated with 2 mM. C&D) cells treated with 1.5 mM.

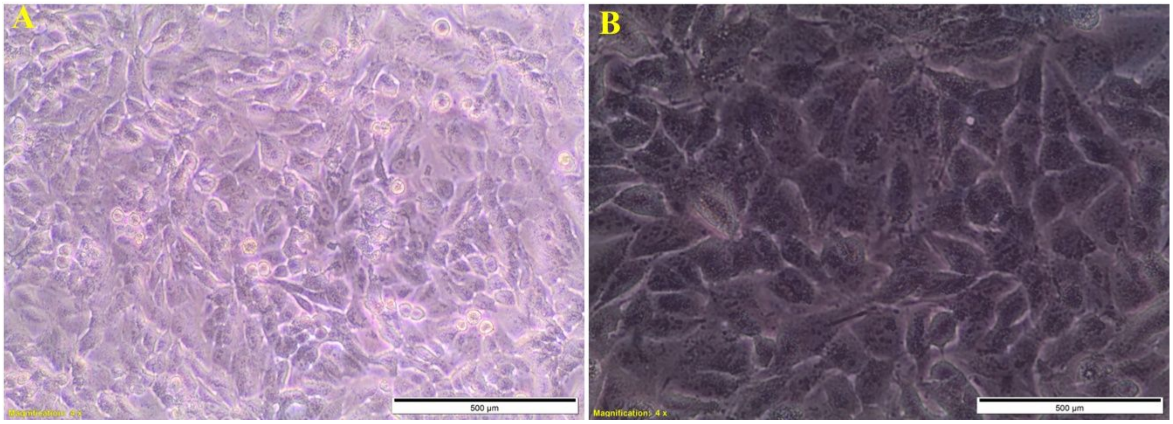

Figure S6. Morphological observation of A549 lung cancer cells without any treatments (untreated control cells). A&B) untreated control cells.

6. Molecular evaluations data

Table S6.1. HULC results (mean and SD)

HSF control	DGN	FUA	CNTCOOHFUADGN	CNTCOOHFUADGN/CSFI.SA

HepG2 control	4.62	0.451	Mean	SD	Mean	SD	Mean	SD	Mean	SD
24 h/0.25 µM			3.17	0.141774	3.2	0.075498	3.153333	0.169214	3.053333	0.075719
24 h/1.75 µM			3.003333	0.10504	2.926667	0.162583	2.953333	0.066583	2.07	0.07
48 h/0.25 µM			1.85	0.15	1.83	0.072111	1.923333	0.051316	1.236667	0.073711
48 h/1.75 µM			1.476667	0.189033	1.433333	0.085049	1.433333	0.195533	1.116667	0.104083

Table S6.2. HOTAIR data

	HepG2		DGN		FUA		CNTCOOHFUADGN		CNTCOOHFUADGN/CSFI.SA	
HepG2 control	14.36667	0.730365	Mean	SD	Mean	SD	Mean	SD	Mean	SD
24 h/0.25 µM			7.88	0.988383	7.633333	0.461772	7.663333	0.506392	6.87	0.645523
24 h/1.75 µM			7.596667	0.612563	7.456667	0.664254	7.466667	0.560922	5.036667	0.151767
48 h/0.25 µM			3.4	0.2	3.326667	0.297041	3.346667	0.235018	3.026667	0.073711
48 h/1.75 µM			2.3	0.1	2.243333	0.222336	2.116667	0.193477	1.35	0.113578

Table S6.3. CCAT-2 data

	HepG2 control		DGN		FUA		CNTCOOHFUADGN		CNTCOOHFUADGN/CSFI.SA	
HepG2 control	2.4	0.1	Mean	SD	Mean	SD	Mean	SD	Mean	SD
24 h/0.25 µM			2.3	0.1	2.243333	0.087369	2.086667	0.133167	1.943333	0.065064
24 h/1.75 µM			1.696667	0.10504	1.626667	0.145717	1.563333	0.080829	1.53	0.101489
48 h/0.25 µM			1.3	0.1	1.33	0.07	1.23	0.08544	1.066667	0.058595
48 h/1.75 µM			1.126667	0.064291	1.176667	0.025166	1.016667	0.028868	1.026667	0.073711

Table S6.4. H19 data

	HepG2 control		DGN		FUA		CNTCOOHFUADGN		CNTCOOHFUADGN/CSFI.SA	
HepG2 control	11.43333	0.46188	Mean	SD	Mean	SD	Mean	SD	Mean	SD
24 h/0.25 µM			4.7	0.360555	4.643333	0.411987	4.556667	0.407717	5.74	0.486621
24 h/1.75 µM			3.433333	0.208167	3.67	0.381182	3.633333	0.345881	4.67	0.376431
48 h/0.25 µM			2.183333	0.225462	2.21	0.181934	2.096667	0.120554	2.316667	0.196044
48 h/1.75 µM			2.033333	0.208167	2.03	0.317648	1.963333	0.165025	1.81	0.091652

Table S6.5. HOTTIP data

	HepG2 control		DGN		FUA		CNTCOOHFUADGN		CNTCOOHFUADGN/CSFI.SA	
HepG2 control	3.666667	0.404145	Mean	SD	Mean	SD	Mean	SD	Mean	SD
24 h/0.25 µM			3.2	0.2	3.203333	0.220076	3.043333	0.1823	3.786667	0.220303
24 h/1.75 µM			2.8	0.264575	2.906667	0.349476	2.766667	0.298719	2.12	0.111355
48 h/0.25 µM			2.083333	0.368556	1.986667	0.232451	1.996667	0.136137	1.87	0.1253
48 h/1.75 µM			1.233333	0.11547	1.153333	0.083865	0.926667	0.080208	1.446667	0.087369

Table S6.1. Mir21 data

	HepG2 control		DGN		FUA		CNTCOOHFUADGN		CNTCOOHFUADGN/CSFI.SA	
HepG2 control	11.7	0.754983	Mean	SD	Mean	SD	Mean	SD	Mean	SD
24 h/0.25 µM			8.833333	0.450925	8.733333	0.448367	8.506667	0.52653	5.866667	0.659571
24 h/1.75 µM			6.9	1.044031	7.013333	0.869617	6.58	1.33	4.313333	0.610765
48 h/0.25 µM			3.2	0.264575	3.186667	0.162583	3.116667	0.150111	2.57	0.387427
48 h/1.75 µM			1.9	0.2	1.893333	0.25658	1.813333	0.223681	1.27	0.288271

Table S6.2. mir92 data

	HepG2 control		DGN		FUA		CNTCOOHFUADGN		CNTCOOHFUADGN/CSFI.SA	
HepG2 control	9.666667	0.90185	Mean	SD	Mean	SD	Mean	SD	Mean	SD
24 h/0.25 µM			5.8	0.556776	5.813333	0.66252	5.586667	0.615819	6.283333	1.031617
24 h/1.75 µM			3.166667	0.305505	3.263333	0.343851	3.063333	0.194251	4.48	0.459021

48 h/0.25 µM	1.8	0.34641	1.823333	0.161967	1.756667	0.299388	2.583333	0.382797
48 h/1.75 µM	0.983333	0.104083	1	0.055678	0.94	0.055678	1.236667	0.230072

Table S6.3. mir145

	HepG2 control		DGN		FUA		CNTCOOHFUADGN		CNTCOOHFUADGN/CSFI.SA	
HepG2 control	0.233333	0.092916	Mean	SD	Mean	SD	Mean	SD	Mean	SD
24 h/0.25 µM			0.5	0.1	0.553333	0.083865	0.563333	0.076376	0.373333	0.106927
24 h/1.75 µM			0.633333	0.057735	0.67	0.101489	0.656667	0.072342	0.66	0.091652
48 h/0.25 µM			0.933333	0.152753	0.99	0.096437	1.09	0.08544	0.79	0.026458
48 h/1.75 µM			1.023333	0.080829	1.06	0.115326	1.023333	0.080829	1.226667	0.111505

Table S6.4. mir 181a

	HepG2 control		DGN		FUA		CNTCOOHFUADGN		CNTCOOHFUADGN/CSFI.SA	
HepG2 control	0.096667	0.015275	Mean	SD	Mean	SD	Mean	SD	Mean	SD
24 h/0.25 µM			0.4	0.1	0.37	0.095394	0.356667	0.076376	0.36	0.075498
24 h/1.75 µM			0.633333	0.057735	0.626667	0.056862	0.596667	0.060277	0.596667	0.145717
48 h/0.25 µM			0.766667	0.066583	0.733333	0.125831	0.763333	0.064291	0.883333	0.070238
48 h/1.75 µM			0.896667	0.015275	0.89	0.05	0.853333	0.045092	1.113333	0.080829

Table S7.1. TGF-B

	HepG2 control		DGN		FUA		CNTCOOHFUADGN		CNTCOOHFUADGN/CSFI.SA	
HepG2 control	174.3333	7.371115	Mean	SD	Mean	SD	Mean	SD	Mean	SD
24 h/0.25 µM			142.6667	1.527525	143	2.645751	141.3333	3.21455	146	28.61818

24 h/1.75 µM	132	3	131.3333	1.527525	130.3333	2.516611	125.3333	8.386497
48 h/0.25 µM	121.3333	2.081666	122.3333	2.081666	121.3333	3.511885	121.3333	1.154701
48 h/1.75 µM	115.6667	5.507571	117.6667	3.511885	115.6667	4.041452	117	1

Table S7.2. E-cadherin

	HepG2 control		DGN		FUA		CNTCOOHFUADGN		CNTCOOHFUADGN/CSFI.SA	
HepG2 control	1.366667	0.057735	Mean	SD	Mean	SD	Mean	SD	Mean	SD
24 h/0.25 µM			1.563333	0.118462	1.546667	0.045092	1.53	0.07	1.55	0.098489
24 h/1.75 µM			1.716667	0.076376	1.766667	0.049329	1.746667	0.060277	1.866667	0.152753
48 h/0.25 µM			2.016667	0.076376	2.103333	0.10504	2.266667	0.057735	2.2	0.1
48 h/1.75 µM			2.466667	0.057735	2.396667	0.092916	2.7	0.087178	2.77	0.060828

[1] S. Ilkar Erdagi, F. Asabuwa Ngwabebhoh, and U. Yildiz, "Genipin crosslinked gelatin-diosgenin-nanocellulose hydrogels for potential wound dressing and healing applications," *International Journal of Biological Macromolecules,* vol. 149, pp. 651-663, 2020/04/15/ 2020, doi: https://doi.org/10.1016/j.ijbiomac.2020.01.279.

[2] S. Das and A. B. H. Wong, "Stabilization of ferulic acid in topical gel formulation via nanoencapsulation and pH optimization," *Scientific Reports,* vol. 10, no. 1, p. 12288, 2020/07/23 2020, doi: 10.1038/s41598-020-68732-6.

[3] S. Wen, H. Liu, H. Cai, M. Shen, and X. Shi, "Targeted and pH-responsive delivery of doxorubicin to cancer cells using multifunctional dendrimer-modified multi-walled carbon nanotubes," (in eng), *Adv Healthc Mater,* vol. 2, no. 9, pp. 1267-76, Sep 2013, doi: 10.1002/adhm.201200389.

[4] M. Anwar, K. Nisa, and N. Indirayati, "Acid-base evaluation of chitosan-ferulic acid conjugate by a free radical grafting method," *IOP Conference Series: Earth and Environmental Science,* vol. 251, p. 012023, 2019/04/12 2019, doi: 10.1088/1755-1315/251/1/012023.

[5] B. Rabha, K. K. Bharadwaj, D. Baishya, T. Sarkar, H. A. Edinur, and S. Pati, "Synthesis and Characterization of Diosgenin Encapsulated Poly-ε-Caprolactone-Pluronic Nanoparticles and Its Effect on Brain Cancer Cells," (in eng), *Polymers (Basel),* vol. 13, no. 8, Apr 18 2021, doi: 10.3390/polym13081322.
